# Mitochondrial transfer-mediated metabolic reprogramming and drug resistance in bone metastasis: mechanisms and therapeutic strategies

**DOI:** 10.3389/fimmu.2026.1863593

**Published:** 2026-07-22

**Authors:** Wei Deng, Bingyu Liu, Songtao Xiang

**Affiliations:** 1 The First Affiliated Hospital of Guangzhou University of Chinese Medicine, Guangzhou, China; 2 The Laboratory of Orthopaedics and Traumatology of Lingnan Medical Research Center, Guangzhou University of Chinese Medicine, Guangzhou, China; 3 Guangzhou University of Chinese Medicine, Guangdong, China

**Keywords:** bone marrow mesenchymal stem cells, bone metastasis, drug resistance, metabolic reprogramming, mitochondrial transfer, oxidative phosphorylation, tumor microenvironment, tunneling nanotubes

## Abstract

Bone is one of the most common sites of distant metastasis in solid tumors, particularly breast cancer, prostate cancer, and lung cancer. Bone metastatic lesions frequently exhibit persistent resistance to multiple systemic therapies, including chemotherapy, targeted therapy, endocrine therapy, and immune checkpoint inhibitors (ICIs). Accumulating evidence suggests that metabolic reprogramming within the bone microenvironment contributes to this resistance, yet the upstream mechanisms remain incompletely understood. Intercellular mitochondrial transfer has emerged as a potential link between the bone marrow niche and tumor metabolic adaptation. Bone marrow mesenchymal stem cells (BMSCs) have been reported to deliver functional mitochondria to tumor cells through tunneling nanotubes (TNTs), extracellular vesicles (EVs), and gap junctions, a process proposed to be regulated by metabolic stress, chemokine signaling (CXCL12/CXCR4), and inflammatory cues. The bone marrow microenvironment—characterized by hypoxia, high cell density, and lipid abundance—together with the intrinsic transfer capacity of BMSCs, may facilitate efficient mitochondrial delivery. Following transfer, exogenous mitochondria have been shown, largely *in vitro* and preclinical models, to restore oxidative phosphorylation (OXPHOS) through respiratory chain reassembly, mitochondrial DNA (mtDNA) replication, and membrane potential recovery, which may drive a metabolic shift from the Warburg phenotype toward a mixed state with enhanced fatty acid β-oxidation (FAO), glutaminolysis, and branched-chain amino acid oxidation. The resulting high adenosine triphosphate (ATP) pool and reshaped redox homeostasis have been proposed to support multiple resistance mechanisms, including ATP-dependent drug efflux, enhanced DNA damage repair, apoptosis resistance, metabolic bypass of targeted therapies, immunosuppressive nutrient competition, and cancer stem cell (CSC) maintenance with therapy-induced dormancy. Mitochondrial dynamics remodeling and metabolic–epigenetic crosstalk may further establish a “metabolic memory” sustaining the resistant phenotype. Notably, transfer patterns appear to exhibit tumor type specificity across breast cancer, prostate cancer, and lung cancer, with multiple myeloma (MM) considered here as a mechanistically informative, marrow-resident comparator rather than a parallel solid-tumor setting. Conceptually proposed therapeutic strategies targeting this axis encompass transfer blockade, OXPHOS and FAO inhibition, and bone-targeted nanodelivery systems, none of which has yet demonstrated efficacy specifically against mitochondrial transfer in patients with bone metastasis. Importantly, several controversies remain unresolved, including the durability and functional integrity of transferred mitochondria, the risk of dye-tracing artifacts, *in vivo* detection sensitivity, and the clinical translation of pathway-specific inhibitors. This review systematically examines the reported molecular mechanisms of mitochondrial transfer between BMSCs and tumor cells, its proposed role in OXPHOS reprogramming and drug resistance in bone metastasis, and emerging therapeutic strategies, aiming to provide a critical framework for developing metabolism-targeted interventions to overcome bone metastatic resistance.

## 1 Introduction

Bone is one of the most common sites of distant metastasis from solid tumors, particularly in breast cancer, prostate cancer, and lung cancer. Epidemiological studies have shown that approximately 70% of patients with advanced or metastatic breast cancer and prostate cancer develop bone involvement, while the incidence of bone metastasis in lung cancer reaches 30%–40% (1). Bone metastasis frequently leads to skeletal-related events (SREs), including pathological fractures, spinal cord compression, and malignant hypercalcemia, which markedly reduce quality of life and are associated with poor prognosis (2). Although bisphosphonates, denosumab, and local radiotherapy can reduce bone destruction and delay skeletal complications to some extent, bone metastasis remains an important factor limiting long-term disease control (3).

Previous studies have largely interpreted the occurrence and maintenance of bone metastasis through the “seed and soil” theory, focusing on the roles of signaling networks such as receptor activator of nuclear factor-κB ligand (RANKL)–RANK and transforming growth factor-β (TGF-β) in tumor colonization and bone remodeling (4). In recent years, with the development of spatial transcriptomics and spatial metabolomics, increasing attention has been directed toward the spatial heterogeneity of metabolic states within bone metastatic lesions. Studies have indicated that tumor cells adjacent to bone marrow regions tend to exhibit higher oxidative phosphorylation (OXPHOS) activity, whereas cells located farther from the marrow show a relatively glycolytic phenotype (5, 6), although these observations are based on a limited number of studies and require independent validation. These observations suggest that, in addition to providing conventional growth factors and cytokines, the bone microenvironment may participate in shaping the metabolic states of tumor cells through metabolic substrate exchange and organelle transfer.

At the clinical level, bone metastatic lesions frequently show persistent low sensitivity to or early progression under various systemic therapies across different tumor types. In hormone receptor (HR)-positive breast cancer, although cyclin-dependent kinase 4/6 (CDK4/6) inhibitors combined with endocrine therapy can substantially prolong progression-free survival (PFS), patients with concurrent bone metastasis tend to show earlier disease progression (7). Similarly, the objective response rate of immune checkpoint inhibitors (ICIs) in bone metastatic lesions is generally lower than that in visceral metastases (8). These clinical observations are consistent with, but do not in themselves prove, the hypothesis that the bone microenvironment may contribute to distinct therapeutic resistance patterns.

Current evidence suggests that metabolic reprogramming represents one of the mechanisms worth further investigation. The bone marrow microenvironment is characterized by hypoxia, lactate accumulation, and lipid abundance, which may induce metabolic plasticity in tumor cells and, under certain conditions, promote a shift toward a relatively enhanced OXPHOS state (9). Within the same bone metastatic lesion, glycolytic, OXPHOS-dominant, and mixed metabolic subpopulations often coexist. Under sustained therapeutic pressure, cells with higher mitochondrial respiratory capacity may gain a survival advantage and contribute to the development of acquired resistance (10). Therefore, inhibition of a single metabolic pathway may not be sufficient to overcome resistance in bone metastasis, and further clarification of the upstream mechanisms underlying the OXPHOS-associated phenotype is of practical importance.

**Figure 1:**
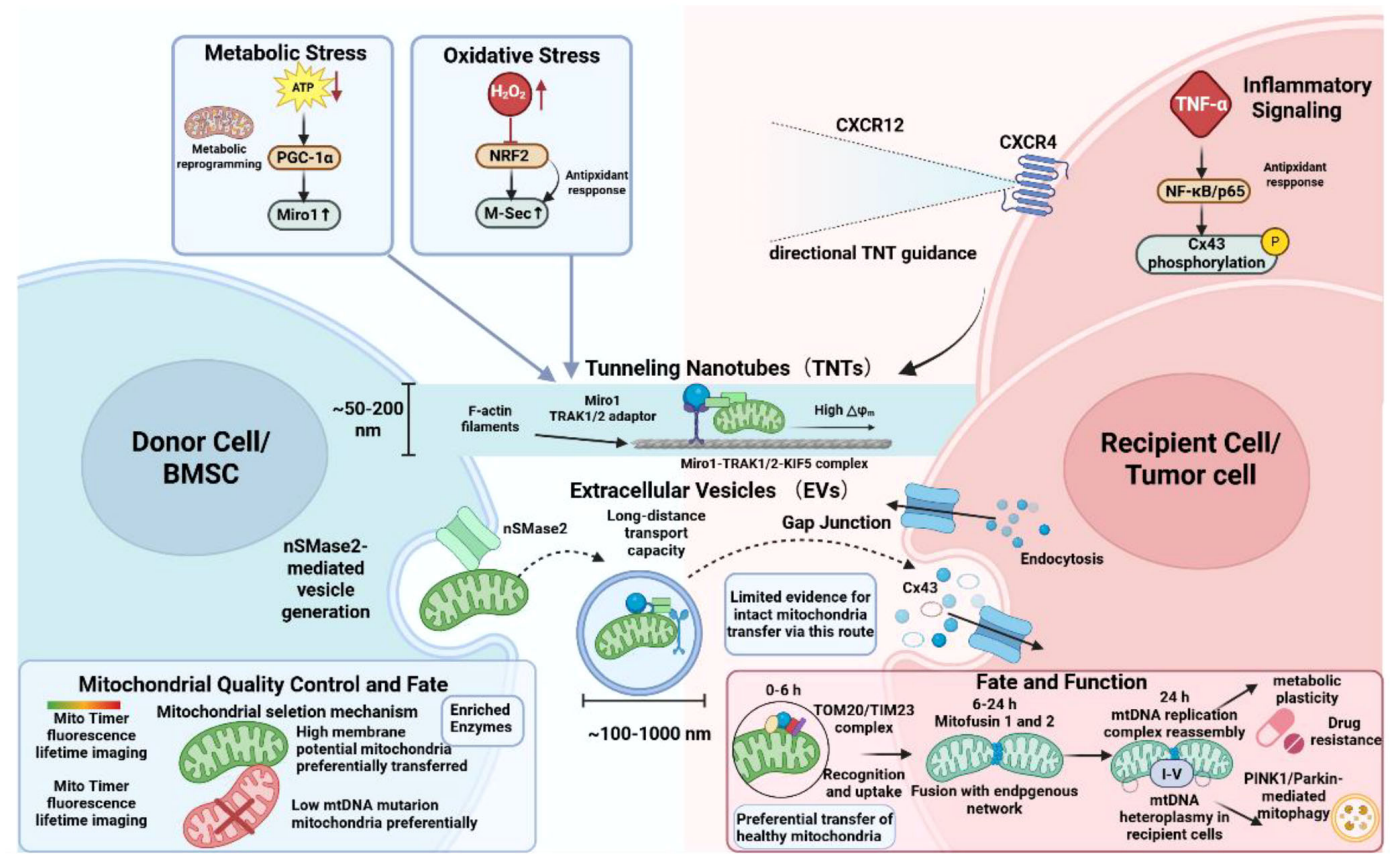
Biological basis of mitochondrial transfer from bone marrow mesenchymal stem cells to tumor cells. This schematic illustrates the molecular pathways, regulatory signals, quality control mechanisms, and post-transfer fate of intercellular mitochondrial transfer within the bone metastatic microenvironment. Donor cells (BMSCs, left) transfer mitochondria to recipient tumor cells (right) through three major routes: tunneling nanotubes (TNTs, ~50–200 nm), mediated by F-actin filaments and the Miro1–TRAK1/2–KIF5 motor complex; extracellular vesicles (EVs, ~100–1000 nm), generated through nSMase2-dependent biogenesis with long-distance transport capacity; and gap junctions via Cx43 channels, for which evidence of intact mitochondrial transfer remains limited. Mitochondrial transfer is triggered by metabolic stress (ATP depletion → PGC-1α → Miro1 upregulation), oxidative stress (H_2_O_2_ → NRF2 → M-Sec upregulation), and inflammatory signaling (TNF-α → NF-κB/p65 → Cx43 phosphorylation) in donor and recipient cells, while the CXCL12/CXCR4 chemokine axis provides directional guidance for TNT extension. A selective packaging mechanism ensures preferential transfer of mitochondria with high membrane potential (ΔΨm) and low mtDNA mutation burden, as revealed by MitoTimer fluorescence lifetime imaging, with enrichment of metabolic enzymes including SOD2 and CPT1A. Following transfer, exogenous mitochondria undergo a sequential integration process: recognition and uptake via the TOM20/TIM23 complex (0–6 h), fusion with the endogenous mitochondrial network mediated by MFN1/2 (6–24 h), and mtDNA replication with respiratory chain complex (I–V) reassembly (>24 h), leading to mtDNA heteroplasmy, enhanced metabolic plasticity, and drug resistance. The functional durability of transferred mitochondria is modulated by PINK1/Parkin-mediated mitophagy in recipient cells.

In this context, intercellular mitochondrial transfer has gradually been incorporated into the discussion of metabolic research on bone metastasis. In 2006, Spees et al. first reported that functional mitochondria can undergo horizontal transfer between mammalian cells (11). Since then, multiple studies in tumor models have shown that bone marrow mesenchymal stem cells (BMSCs) can transfer mitochondrial components or relatively intact mitochondrial structures to tumor cells through tunneling nanotubes (TNTs) or extracellular vesicles (EVs), thereby enhancing mitochondrial respiratory function in recipient cells to some extent (12, 13). Mitochondrial transport-related molecules such as mitochondrial Rho GTPase 1 (Miro1) have been implicated in the regulation of this process (**Figure 1**) (14). In several preclinical models of bone metastasis, inhibition of mitochondrial transfer between BMSCs and tumor cells has been shown to increase tumor cell sensitivity to chemotherapy (15).

However, consistent evidence regarding the frequency, duration, and actual contribution of this process to resistance development *in vivo* remains limited. Current studies have not reached consensus on whether exogenous mitochondria can be stably retained and continuously participate in the metabolic activities of recipient cells (16, 17), and the sensitivity and specificity of *in vivo* quantitative tracking methods remain limited (18). It should also be noted that much of the evidence supporting the role of mitochondrial transfer in bone metastatic resistance is derived from hematologic malignancies and *in vitro* co-culture systems; direct *in vivo* evidence in solid-tumor bone metastasis remains comparatively limited and warrants careful interpretation.

Previous reviews of mitochondrial transfer have primarily addressed its general biological mechanisms or its role in non-malignant pathophysiology such as cardiac and neurological diseases. The present review focuses specifically on the bone metastatic microenvironment, with three distinguishing features: (1) it explicitly differentiates evidence derived from marrow-resident hematologic malignancies, *in vitro* systems, and solid-tumor bone metastasis; (2) it provides a critical synthesis rather than a mechanistic catalog, with explicit attention to evidence strength, controversies, and unresolved questions; and (3) it proposes a conceptual framework linking mitochondrial transfer, OXPHOS-centered metabolic reprogramming, and bone metastasis–specific therapeutic resistance, while acknowledging the preclinical and exploratory nature of much of the supporting evidence. 

## 2 Biological basis of mitochondrial transfer

Mitochondrial transfer generally refers to the process by which donor cells deliver functionally active mitochondria to recipient cells, thereby influencing the metabolic state of the latter (17).At present, the identification of this process emphasizes the integration of structural, functional, and spatiotemporal evidence; that is, the actual transfer of mitochondrial components or structures, alterations in oxidative phosphorylation (OXPHOS) function in recipient cells, and a clearly defined transfer relationship between donor and recipient cells must be demonstrated simultaneously (19, 20). Compared with the transfer of mitochondrial DNA (mtDNA) or mitochondrial fragments alone, the transfer of intact mitochondria is more likely to exert substantial effects on recipient cell respiratory function (21, 22). In recent years, parameters such as membrane potential, oxygen consumption rate (OCR), and mtDNA changes have been progressively introduced to quantitatively evaluate mitochondrial transfer, thereby advancing the field from descriptive observation toward functional validation and methodological standardization.

### 2.1 Major molecular pathways of mitochondrial transfer

Mitochondrial transfer is primarily mediated through tunneling nanotubes (TNTs), extracellular vesicles (EVs), and gap junctions, all of which may participate in intercellular metabolic exchange within the bone metastatic microenvironment (16). TNTs are F-actin–dependent membranous structures that mediate directional mitochondrial transport between adjacent cells. Mitochondrial Rho GTPase 1 (Miro1) and its associated motor protein complexes are considered to participate in the regulation of this process,and the bone marrow chemotactic signal C-X-C motif chemokine ligand 12/C-X-C motif chemokine receptor 4 (CXCL12/CXCR4) axis has also been shown to promote TNT formation (23, 24).

EVs have been proposed as a route for longer-distance transfer. Vesicle biogenesis and mitochondrial component loading mediated by neutral sphingomyelinase 2 (nSMase2) have been associated with partial restoration of respiratory function in recipient cells in selected studies (25); however, whether EV cargo includes intact functional mitochondria or predominantly mitochondrial fragments and proteins remains controversial. Gap junctions are thought to mediate the exchange of small molecules or mitochondria-associated components through connexin 43 (Cx43)-containing channels, while direct evidence for the transfer of intact mitochondria via this pathway is comparatively limited (26). Comparative studies have suggested that TNTs may exhibit higher transfer efficiency, while EVs may offer advantages in transfer distance; these conclusions are based largely on *in vitro* comparisons and have not been systematically validated *in vivo* in bone metastasis settings. The high density of intercellular connections within the bone marrow microenvironment has been hypothesized to further facilitate these processes (13).

### 2.2 Triggering signals and regulatory networks of mitochondrial transfer

Mitochondrial transfer has been reported to be regulated by multilevel stress signals, involving interactions among metabolic stress, oxidative stress, chemokines, and inflammatory signaling pathways. At the level of metabolic stress, a decline in adenosine triphosphate (ATP) levels can activate peroxisome proliferator-activated receptor γ coactivator-1α (PGC-1α), which promotes TNT formation through transcriptional upregulation of Miro1, whereas knockdown of PGC-1α can block the majority of transfer events (27). In addition, a CXCL12 concentration gradient can guide the directional extension of TNTs (see Section 2.2), and this effect can be reversed by CXCR4 antagonists (28). Furthermore, in the inflammatory microenvironment, tumor necrosis factor-α (TNF-α) can activate Cx43 phosphorylation through the nuclear factor-κB (NF-κB)/p65 pathway, thereby enhancing gap junction–mediated mitochondrial transfer.

Mitochondrial transfer appears not to occur randomly but to involve selective packaging mechanisms, based on findings from a limited number of imaging studies. Using the MitoTimer fluorescence lifetime imaging system, donor cells have been reported to preferentially transfer mitochondria with higher membrane potential and lower mtDNA mutation burden, with transfer efficiencies substantially higher than those of damaged mitochondria. Single-mitochondrion mass spectrometry analysis has also reported enrichment of metabolic enzymes such as superoxide dismutase 2 (SOD2) and carnitine palmitoyl transferase 1A (CPT1A) in transferred mitochondria (29). These findings are derived from a limited number of single-cell-level studies and await broader independent validation. Within the bone metastatic microenvironment, hypoxia and high lactate conditions have been proposed to synergistically activate the hypoxia-inducible factor-1α (HIF-1α)/Miro1 signaling axis, further enhancing mitochondrial transfer efficiency (30). Time-resolved transcriptomic analysis has further revealed that mitochondrial transfer peaks between 24 and 48 hours after chemotherapy, closely coinciding with the time window of resistant clone expansion (31). The clinical relevance of this temporal window in human patients remains to be established, although it has been proposed as a potential target window for intervention.

### 2.3 Fate and functional integration of transferred mitochondria

After entering recipient cells, exogenous mitochondria have been proposed to undergo multiple stages, including recognition, fusion, and functional restoration, before effective integration can be achieved. Single-mitochondrion tracking studies have provided a temporal characterization of this process in cell culture systems: within 0 to 6 hours post-transfer, the translocase of the outer membrane 20/translocase of the inner membrane 23 (TOM20/TIM23) complex has been reported to mediate the recognition and uptake of exogenous mitochondria; between 6 and 24 hours, mitofusin 1 and 2 (MFN1/2) have been reported to drive fusion of exogenous mitochondria with the endogenous mitochondrial network, forming a hybrid network; after 24 hours, mtDNA replication has been reported to be initiated, with progressive reassembly of respiratory chain complexes and restoration of OXPHOS function (32, 33). It should be emphasized that this temporal sequence has been characterized predominantly in *in vitro* systems, and whether comparable kinetics occur *in vivo* within bone metastatic lesions remains unverified.

At the genetic level, donor mtDNA introduced through transfer has been reported to be amplified within recipient cells and to form stable mtDNA heteroplasmy in some experimental settings (34). This mtDNA heterogeneity has been proposed to confer greater metabolic plasticity, and cells carrying mixed mtDNA populations have been reported to exhibit enhanced tolerance to metabolic inhibitors. However, findings regarding the functional durability of transferred mitochondria are inconsistent and remain a central controversy in the field: some studies have reported that exogenous mitochondria can maintain function for up to three weeks (35),whereas others have observed that most transferred mitochondria undergo degradation within one week. Further analysis has suggested that this discrepancy may be related to the level of PTEN-induced kinase 1/Parkin (PINK1/Parkin)-mediated mitophagy in recipient cells — recipient cells with high mitophagic activity tend to degrade exogenous mitochondria, whereas those with low mitophagic activity may achieve longer-term integration. A second source of uncertainty is whether the transferred material in these studies represents intact functional organelles or fragments and components that mimic full mitochondrial transfer when assessed by fluorescent labels alone. These unresolved issues directly affect interpretation of the biological significance of mitochondrial transfer and should be considered when evaluating mechanistic claims throughout the literature.

Regarding metabolic function, carbon-13 (¹³C)-labeled fatty acid tracing studies have reported that transferred mitochondria preferentially activate the fatty acid β-oxidation (FAO) pathway over the glycolytic pathway, with FAO contributing a relatively high proportion of ATP production in these experimental systems (36). These observations suggest that autophagy regulation may play a dual role in determining the fate of transferred mitochondria, although the precise balance between degradation and integration remains to be defined (37).

### 2.4 Detection and quantification techniques for mitochondrial transfer

Accurate detection and quantification of mitochondrial transfer represent critical technical challenges in this field. Existing methods differ in their specificity, sensitivity, and applicable scenarios, and no single approach has yet emerged as a fully validated standard for *in vivo* studies in bone metastasis.

The mtDNA barcode tracing technique, currently regarded as the gold standard, employs clustered regularly interspaced short palindromic repeats/CRISPR-associated protein 9 (CRISPR/Cas9) to edit and introduce unique nucleotide barcode sequences into donor cell mtDNA, followed by quantitative polymerase chain reaction (qPCR) detection of donor-derived mtDNA in recipient cells. *In vivo* validation has demonstrated that this technique can precisely detect mitochondrial transfer from BMSCs to tumor cells in mouse bone metastasis models (38). Genetic tracing methods based on the Cre-LoxP system offer high specificity and have been applied in several *in vivo* studies, predominantly in hematologic malignancy models. The photoconvertible fluorescent protein Mito-Dendra2 is suitable for live imaging experiments requiring high spatiotemporal resolution, enabling dynamic tracking of mitochondrial transport *in vitro* and in selected *in vivo* settings.

In contrast, MitoTracker dye labeling combined with flow cytometry is relatively simple and cost-effective but is subject to well-recognized technical limitations: dye leakage between cells, transfer of stained membrane fragments unrelated to functional mitochondrial transfer, and uptake of cellular debris can all generate false-positive signals that inflate apparent transfer frequencies (39). At the level of functional assessment, the functional mitochondrial transfer index (FTI) combined with Seahorse XF metabolic analysis has been proposed as a means to distinguish functional from non-functional transfer events.

Recent studies have further developed a dual-photon microscopy and mtDNA barcode combined detection system, enabling real-time visualization of TNT formation and mitochondrial transport dynamics in bone metastatic lesions of the mouse tibia (40). In terms of clinical translation, positron emission tomography (PET) imaging using ¹^8^F-labeled mitochondrial uptake probes has been reported in early preclinical work, with probe uptake values in bone metastatic lesions showing correlation with FTI in a limited number of animal studies (41). This approach remains exploratory and has not yet been validated in human studies. Taken together, the integration of multimodal detection approaches — including mtDNA barcode tracing, FTI functional assessment, and molecular imaging — may help establish a more standardized framework for mitochondrial transfer detection. At present, however, methodological heterogeneity remains a major obstacle to comparing findings across studies and to assessing the true frequency of mitochondrial transfer in clinically relevant settings.

### 2.5 Major unresolved controversies in mitochondrial transfer biology

The mechanisms outlined in Sections 2.1 to 2.4 have advanced considerably over the past decade, yet several foundational questions remain unresolved. These controversies are not peripheral; they directly shape how the experimental evidence summarized in subsequent sections should be interpreted, and we therefore highlight five recurring issues before proceeding to the discussion of specific biological contexts.

A primary unresolved question concerns the nature of the material being transferred between cells. Studies based on fluorescent membrane labels or mitochondria-targeted dyes have frequently been interpreted as evidence of intact organelle transfer, yet the same signals may also arise from membrane fragments, mitochondrial-derived vesicles, or mtDNA released from damaged organelles, and electron microscopy confirmation of intact double-membraned structures in recipient cells remains comparatively scarce (17, 42). This distinction is biologically meaningful, because intact mitochondria can in principle support sustained OXPHOS, whereas isolated fragments may at best contribute short-term metabolites, mtDNA copies, or selected proteins (43). A closely related controversy concerns the durability of transferred mitochondria once inside recipient cells, with reports of functional persistence ranging from approximately one week to three weeks and some studies suggesting that the majority of exogenous mitochondria are degraded by PINK1/Parkin-mediated mitophagy within a relatively short timeframe (44, 45) (Section 2.3). Short-lived events may provide only transient metabolic buffering, whereas durable integration would be required to support the longer-term phenotypes — sustained OXPHOS reprogramming, drug resistance, stemness maintenance — that are frequently attributed to mitochondrial transfer.

A third concern is methodological. A substantial proportion of the early literature relied on dye-based labels such as MitoTracker derivatives, which are known to leak between cells, transfer with non-mitochondrial membrane debris, and produce false-positive signals in flow cytometry assays; more recent approaches based on mtDNA barcoding, Cre-LoxP genetic tracing, and photoconvertible Mito-Dendra2 reporters have improved specificity but remain technically demanding, and dye-based frequency estimates from earlier studies should be weighed accordingly (46, 47). Linked to this is a fourth issue, namely whether the transfer frequencies reported in experimental systems correspond to physiologically relevant levels: many *in vitro* co-culture studies report transfer to a substantial fraction of recipient cells within 24–48 hours, whereas *in vivo* studies generally report considerably lower frequencies confined to small subsets of tumor cells in spatially defined niches, and high donor-to-recipient cell ratios, prolonged direct contact, and metabolic stress *in vitro* may artificially amplify events that occur only sporadically under physiological conditions (48, 49).

The fifth and most translationally important controversy is whether mitochondrial transfer meaningfully contributes to therapeutic resistance in human patients with bone metastasis. Most mechanistic evidence is derived from *in vitro* co-culture systems or murine preclinical models, with relatively few studies analyzing human bone metastatic specimens directly; correlative human data are suggestive but do not establish causation, no prospective clinical study has yet quantified the contribution of mitochondrial transfer to treatment failure in human bone metastasis, and no clinical trial has formally evaluated interventions designed to block this process in patients (11, 50). These five controversies should be kept in mind throughout the subsequent sections (16). Where the evidence base is thin or methodologically heterogeneous, we have used hedged language and explicit qualifiers rather than definitive statements, an approach that we believe better reflects the current state of the field and provides a more useful framework for identifying research gaps.

## 3 The unique bone metastatic microenvironment

### 3.1 Cellular composition and function of the bone microenvironment

The bone marrow microenvironment comprises multiple cell populations, including hematopoietic lineage cells, mesenchymal lineage cells, and immune cells, which together form an ecological network that has been proposed to support tumor colonization and survival. Among these, BMSCs have been described as the principal stromal support cells. BMSCs maintain the hematopoietic stem cell niche through CXCL12 secretion and have been reported to transfer mitochondria to tumor cells via TNTs and other routes (see Section 2.1) (5). The density of BMSCs within bone metastatic lesions has been reported to be substantially higher than that in normal bone marrow in selected studies, and upregulation of Miro1 expression in BMSCs has been associated with increased transfer efficiency (51).

At the level of bone remodeling, osteoblasts and osteoclasts constitute a dynamically balanced functional unit. Osteoclasts release growth factors stored in the bone matrix, such as TGF-β and insulin-like growth factor 1 (IGF-1), through bone resorption, contributing to the classical “vicious cycle” (4). Single-cell RNA sequencing (scRNA-seq) studies have suggested that metabolites produced by osteoclasts may be transported to tumor cells via monocarboxylate transporter 1 (MCT1), potentially contributing to the regulation of tumor metabolic phenotype (52). Osteoblasts, in turn, secrete osteoprotegerin (OPG) to inhibit RANKL signaling and express Cx43, which may support gap junction–mediated material exchange (53).

In addition to the cell types described above, bone marrow adipocytes (BMAds) have been proposed to play a dual role in bone metastasis — providing fatty acids as metabolic substrates for tumor cells (discussed in detail in Section 3.3) and secreting parathyroid hormone-related protein (PTHrP), which has been implicated in bone microenvironment remodeling (54). EVs derived from BMAds have been reported to contain mitochondrial components and to be delivered to tumor cells in *in vitro* studies (12). Regarding immune cells, tumor-associated macrophages (TAMs) and myeloid-derived suppressor cells (MDSCs) have been reported to activate the mitochondrial transfer program of BMSCs through interleukin-6 (IL-6)/TNF-α signaling in preclinical models (55). In summary, multiple cell types within the bone microenvironment may form a multilayered tumor-supportive network through paracrine signaling, metabolite transport, and direct cell–cell contact. The role of BMSCs in mediating mitochondrial transfer has positioned them as a potential intervention target warranting further investigation (56), though direct evidence from solid-tumor bone metastasis remains less abundant than from hematologic malignancy contexts.

### 3.2 Metabolic extension of the vicious cycle hypothesis

The classical vicious cycle hypothesis describes a positive feedback mechanism in which tumor cell–secreted PTHrP activates osteoclasts, and the subsequent bone resorption releases TGF-β, which in turn promotes tumor growth (57). Recent studies have extended this framework by revealing a metabolic dimension of the cycle: osteoclast-mediated bone resorption releases not only growth factors but also triglycerides (TGs) and free fatty acids (FFAs), providing metabolic substrates for tumor cells (58). Local lactate concentrations at bone resorption sites are also substantially elevated and can be unidirectionally transported to tumor cells via monocarboxylate transporter 4 (MCT4) to sustain their glycolytic flux (59). Concurrently, tumor cell–secreted RANKL activates osteoclasts, which depend on vacuolar-type H^+^-ATPase (V-ATPase) to maintain the acidic environment required for bone resorption (60).

At the level of metabolic coupling, ¹³C-glucose tracing studies have identified a lactate shuttle between tumor cells and osteoblasts—lactate produced by tumor cells is converted to pyruvate by osteoblast lactate dehydrogenase (LDH) to support OXPHOS (61, 62). Under hypoxic conditions, HIF-1α signaling activation (see Section 2.2) upregulates glucose transporter type 1 (GLUT1) in tumor cells, enabling them to compete with osteoblasts for glucose and thereby suppressing bone formation (63). Mitochondrial transfer has also been integrated into this metabolic circuit: elevated reactive oxygen species (ROS) induced by osteoclast stress can activate mitochondrial transfer from BMSCs to tumor cells, forming a positive feedback loop of “bone resorption–mitochondrial transfer–tumor survival” (14).

These findings suggest that the vicious cycle of bone metastasis may be driven by both signaling and metabolic mechanisms, and that combined targeting of signaling and metabolic pathways may hold synergistic therapeutic potential (8). The metabolic extension of the vicious cycle, however, is largely supported by preclinical models and remains to be validated in human bone metastasis specimens.

### 3.3 Metabolic characteristics of the bone microenvironment

The bone marrow microenvironment possesses a distinct metabolic landscape that provides conditions for metabolic plasticity transitions in tumor cells. Hypoxia (1–5% O_2_), an inherent feature of the bone marrow, regulates the metabolic balance between glycolysis and OXPHOS through HIF-1α and related signaling pathways (see Section 2.2) (64). Spatial metabolic imaging studies have reported the presence of distinct metabolic zonation within bone metastatic lesions: tumor cells in the cancellous bone region tend to exhibit an OXPHOS-dependent phenotype, whereas those in the cortical bone region appear to maintain a glycolytic phenotype (65). These spatial patterns are based on a limited number of imaging studies and require independent confirmation.

Regarding cellular properties, BMSCs express high levels of Miro1 and PGC-1α, and their mtDNA copy number is substantially higher than that of mesenchymal stem cells from other tissue sources, endowing them with an inherently strong capacity for mitochondrial transfer (66, 67). Hypoxia and high calcium levels can synergistically activate the mitochondrial biogenesis program in BMSCs, with the mitochondrial calcium uniporter (MCU)–Ca²^+^ axis upregulating the expression of related genes (68). ATP efflux from tumor cells following chemotherapy damage has been reported to activate the purinergic receptor P2Y2 on BMSCs, which has been associated with directional TNT growth (69).

Anatomical features also contribute to enhanced transfer efficiency. The spatial architecture of the bone marrow cavity facilitates sustained cell–cell contact. Endothelial cells lining Haversian canals express intercellular adhesion molecule 1 (ICAM-1), which mediates the adhesion of tumor cells to BMSCs (70). It should be emphasized, however, that several of these features — including the proposed intrinsic transfer capacity of BMSCs and the higher transfer frequency in bone versus lung metastases (71)-derive from a small number of studies that have not been independently replicated, and their relative contributions in human bone metastatic lesions remain unquantified. Therapeutic strategies targeting bone metastasis may need to take these multidimensional factors into consideration (72).

### 3.4 Biological distinctions between hematologic malignancies and solid-tumor bone metastasis

A substantial portion of the mitochondrial transfer literature relevant to this review is derived from hematologic malignancy models, particularly acute myeloid leukemia (AML) and multiple myeloma (MM), rather than from solid-tumor bone metastasis. Because these settings share the bone marrow as a common anatomical location, mechanistic findings from hematologic malignancies have frequently been extrapolated to solid-tumor bone metastasis, sometimes without explicit consideration of the underlying biological differences (73, 74). This section examines what can and cannot be transferred between these settings, and identifies where conclusions remain insufficiently supported in the solid-tumor bone metastasis context that forms the principal focus of this review.

Hematologic malignancies and solid-tumor bone metastases occupy the bone marrow under fundamentally different biological circumstances (75–77). AML and MM arise within the marrow as their primary niche, so that malignant cells exist in close physical contact with BMSCs from disease onset, with an evolutionary history of co-adaptation to the marrow environment (78). Solid tumor cells, by contrast, originate in distant primary organs and reach the bone marrow only after a multistep process of intravasation, circulation, extravasation, and colonization, arriving as relatively small populations that must establish contact with BMSCs from a foreign starting state (79, 80). This difference has direct consequences for the interpretation of transfer data, because the high transfer frequencies frequently reported in MM and AML models likely reflect not only intrinsic donor–recipient compatibility but also the high local density of malignant cells, their prolonged contact with BMSCs, and adaptive remodeling of the niche over weeks to months of disease evolution; disseminated solid tumor cells, particularly during early colonization, encounter the marrow as comparatively transient guests, and transfer frequencies in this setting cannot be assumed to match those observed in primary marrow malignancies (81, 82).

The bone microenvironmental phenotype induced by different malignancies further differentiates these contexts. Breast and lung cancer bone metastases are predominantly osteolytic, dominated by osteoclast hyperactivation and matrix degradation that releases stored growth factors and lipids (1, 83); prostate cancer bone metastases are predominantly osteoblastic, with dense osteoblast networks and a distinct cytokine milieu (84); AML and MM, in contrast, occupy the marrow compartment itself, with more diffuse and stage-dependent effects on bone remodeling (85). Each phenotype establishes a different cellular composition, a different set of dominant donor cells for potential mitochondrial transfer, and different metabolic substrate availability, so that conclusions about which donor cells matter and which transfer routes dominate cannot be uniformly extrapolated across these settings.

Some categories of evidence nevertheless appear reasonably transferable. The structural basis of TNTs, the function of Miro1 and associated motor proteins, the role of nSMase2 in EV biogenesis, and Cx43-mediated gap junction communication are conserved cellular processes whose general features are unlikely to differ qualitatively between disease backgrounds (17, 86), and the basic observation that recipient cells can show enhanced OXPHOS-related parameters after mitochondrial acquisition has been replicated across multiple cell types and disease models (13, 87). By contrast, quantitative transfer frequencies, niche residency dynamics, disease stage–specific transfer patterns, the coupling between transfer and bone remodeling, and the specific resistance pathways involved — for instance, bortezomib resistance in MM versus endocrine or targeted therapy resistance in solid-tumor bone metastasis — do not transfer well between contexts and require independent investigation in solid-tumor bone metastasis models (88, 89).

For solid-tumor bone metastasis specifically, the directly applicable evidence base is more limited than the hematologic malignancy literature might suggest. The best-developed evidence concerns breast cancer, drawing predominantly from *in vitro* co-culture systems and selected murine bone metastasis models (Sections 3.1 and 7.1) (90), and prostate cancer, drawing from single-cell mtDNA sequencing and co-culture experiments (Section 7.2) (18), with evidence in lung cancer bone metastasis remaining the least developed (Section 7.3) (91, 92). Direct visualization of mitochondrial transfer in human bone metastatic specimens remains rare (93), quantitative cross-tumor comparisons in patient-derived models have not been performed systematically, and prospective clinical evidence linking mitochondrial transfer markers to treatment response in solid-tumor bone metastasis is lacking (94, 95). In the subsequent sections, we have indicated where possible whether specific findings derive from hematologic malignancy systems, solid-tumor bone metastasis models, *in vitro* co-culture, or clinical specimens, and we discuss MM in Section 6.4 as a mechanistically informative but biologically distinct comparator rather than as a parallel disease setting.

## 4 Evidence and patterns of mitochondrial transfer in bone metastasis

### 4.1 Mitochondrial transfer from BMSCs to tumor cells

Following mitochondrial transfer from BMSCs to MCF-7 cells via TNTs (see Section 2.1) in co-culture systems, respiratory chain complex activity has been reported to be restored and survival capacity enhanced in recipient cells. In a murine model of breast cancer bone metastasis, BMSCs have been reported to transfer mitochondria to MCF-7 cells through TNTs, with restoration of respiratory chain complex activity and enhanced cell survival. MitoTracker tracing has indicated that the transferred mitochondria may maintain function in recipient cells for up to two weeks (51, 96), although interpretation of MitoTracker-based duration data is subject to the technical caveats discussed in Section 2.4.

In hematological malignancies, *in vivo* studies using the Cre-loxP system have confirmed that mitochondrial transfer from BMSCs to leukemia cells occurs within the acute myeloid leukemia (AML) bone marrow niche, and this transfer is significantly associated with post-chemotherapy relapse risk. Blockade of CXCL12/CXCR4-dependent transfer (see Section 1.1) with the CXCR4 antagonist AMD3100 restored the sensitivity of leukemia cells to cytarabine (14). It should be emphasized that AML is a marrow-resident hematologic malignancy whose direct contact with BMSCs is biologically distinct from the colonization process of disseminated solid tumors; therefore, while AML evidence supports the general principle of BMSC–tumor mitochondrial transfer, direct extrapolation of mechanisms and frequencies to solid-tumor bone metastasis requires caution.

Studies on prostate cancer bone metastasis have provided supporting evidence in a solid-tumor context: single-cell mtDNA sequencing has reported that PC3 cells acquire mitochondria from adjacent BMSCs, with increases in mtDNA heteroplasmy temporally associated with development of androgen deprivation therapy (ADT) resistance. *In vitro* co-culture experiments have indicated that transfer efficiency is positively correlated with BMSC number (97, 98), though large-scale *in vivo* validation in patient-derived models remains limited.

Additionally, multiple myeloma (MM), as a primary bone marrow malignancy, has provided relatively direct evidence: MM.1S cells that acquire mitochondria from BMSCs have been reported to show restored OXPHOS flux and reduced bortezomib-induced apoptosis (99). As with AML, however, MM resides within the bone marrow as its primary niche, and its relationship with BMSCs is intrinsically different from that of metastatic solid tumors that colonize bone secondarily.

Taken together, these findings across multiple tumor types support the concept that mitochondrial transfer from BMSCs to tumor cells may serve as one mechanism by which the bone marrow niche supports tumor metabolic adaptation. The strength of evidence varies considerably across tumor types: *in vivo* evidence is most extensive for AML and MM, intermediate for breast cancer and prostate cancer bone metastasis, and largely confined to early co-culture or model studies for lung cancer bone metastasis. Specific blockade of this process has been proposed as a candidate strategy for overcoming resistance in bone metastasis, but clinical evidence in humans is currently lacking (87).

### 4.2 Mitochondrial transfer patterns between other cell types

In addition to BMSCs, multiple cell types within the bone microenvironment participate in mitochondrial transfer, forming a multi-source metabolic support network. BMAds can transfer mitochondria to breast cancer cells via EVs, with the fatty acid binding protein 4/cluster of differentiation 36 (FABP4–CD36) axis mediating concomitant fatty acid transfer, thereby enhancing FAO flux and survival capacity of tumor cells under nutrient-restricted conditions (12). Osteoblasts support the co-transfer of small molecules such as mitochondrial transfer RNA (mt-tRNA) through Cx43 hemichannels and can transfer mitochondria to tumor cells under osteoclast stress conditions to maintain OXPHOS function (100).

Osteoclasts have been proposed to participate in transfer through a distinct mechanism. Chemotherapy-damaged osteoclasts release mitochondrial fragments that have been reported to be taken up by tumor cells via stabilin-2 receptor–mediated endocytosis. Notably, these fragments are structurally incomplete, and whether they meaningfully restore respiratory function in recipient cells — as opposed to providing only short-term metabolic intermediates — remains under investigation (101). Immune cells are also involved in this transfer network: TAMs can acquire mitochondria from BMSCs and subsequently relay them to tumor cells, forming a cascade transfer chain (13). MDSCs have been reported to transfer mitochondria to regulatory T cells (Tregs) through TNTs, an interaction associated with enhanced immunosuppressive function (102). It should be noted that several of these inter-cellular transfer observations (including TAM- and MDSC-related events) derive predominantly from non-bone or non–solid-tumor model systems, and their occurrence within solid-tumor bone metastatic lesions has not been directly demonstrated. Much of this multi-cellular transfer evidence is derived from individual studies and has not been independently reproduced; the relative quantitative contribution of each donor population *in vivo* remains largely unquantified. The apparent multi-cellular origin of mitochondrial transfer suggests that intervention strategies targeting a single donor cell type may have limited efficacy, although this hypothesis itself requires direct testing.

### 4.3 Spatiotemporal dynamics of mitochondrial transfer in bone metastasis

Mitochondrial transfer has been reported to follow distinct spatiotemporal patterns during the progression of bone metastasis in preclinical models. *In vivo* dual-photon microscopy tracking has reported that mitochondrial transfer begins during the niche formation phase following tumor cell colonization (approximately days 3–7), peaks during the micrometastasis formation phase (approximately days 14–28), and decreases during the dormancy phase, although it has been reported to be rapidly upregulated in response to therapeutic stress (103). These temporal patterns are based on a limited number of murine imaging studies and have not been validated in patient-derived models or human specimens.

Regarding spatial distribution, the transfer frequency in cancellous bone regions is substantially higher than in cortical bone regions. Spatial transcriptomic analysis has indicated that this difference is associated with higher BMSC density and a denser TNT network in cancellous bone regions (104). Differences also exist between proximal long bones and axial skeletal sites, with endothelial cells lining Haversian canals in the former expressing high levels of ICAM-1 to promote adhesion between tumor cells and BMSCs (105).

Therapeutic pressure can substantially reshape mitochondrial transfer dynamics. Transfer frequency increases markedly within 24 hours after chemotherapy and remains elevated (106), compatible with the observation of a 24- to 48-hour post-chemotherapy transfer peak window described in Section 2.2. Metabolic stress induced by targeted therapy can also activate the transfer program; for example, mitochondrial transfer events are substantially increased in epidermal growth factor receptor (EGFR)-mutant lung cancer bone metastatic lesions following osimertinib treatment (107). At the single-cell level, analysis using the Mito-Dendra2 photoconversion system at single-cell resolution has revealed that individual tumor cells can simultaneously acquire mitochondria from multiple BMSCs, exhibiting a multi-source convergence pattern (108).

These spatiotemporal features provide a reference for the design of intervention strategies: the niche formation phase may represent a potential window for preventive intervention; the post-treatment transfer peak may be a critical period for combination therapy; and the spatial heterogeneity of transfer distribution may inform the optimization of local delivery strategies.

### 4.4 Key factors influencing mitochondrial transfer efficiency

Mitochondrial transfer efficiency is subject to multilevel regulation by donor cells, recipient cells, and microenvironmental factors. At the donor cell level, the quality and quantity of mitochondria in BMSCs directly affect transfer efficiency. BMSCs with higher mtDNA copy numbers and stable membrane potential exhibit substantially greater transfer efficiency than aged or functionally impaired BMSCs (109). PGC-1α, as the principal regulator of mitochondrial biogenesis, determines the mitochondrial transfer capacity of donor cells through its expression level (110).

At the recipient cell level, the degree of mitochondrial damage in tumor cells is positively correlated with the efficiency of exogenous mitochondrial uptake. Elevated intracellular ROS levels are accompanied by increased transfer frequency (111), and expression of the phosphatidylserine receptor T-cell immunoglobulin mucin receptor 1 (Tim-1) on the recipient cell surface can enhance the recognition and uptake of exogenous mitochondria (112).

**Figure 2:**
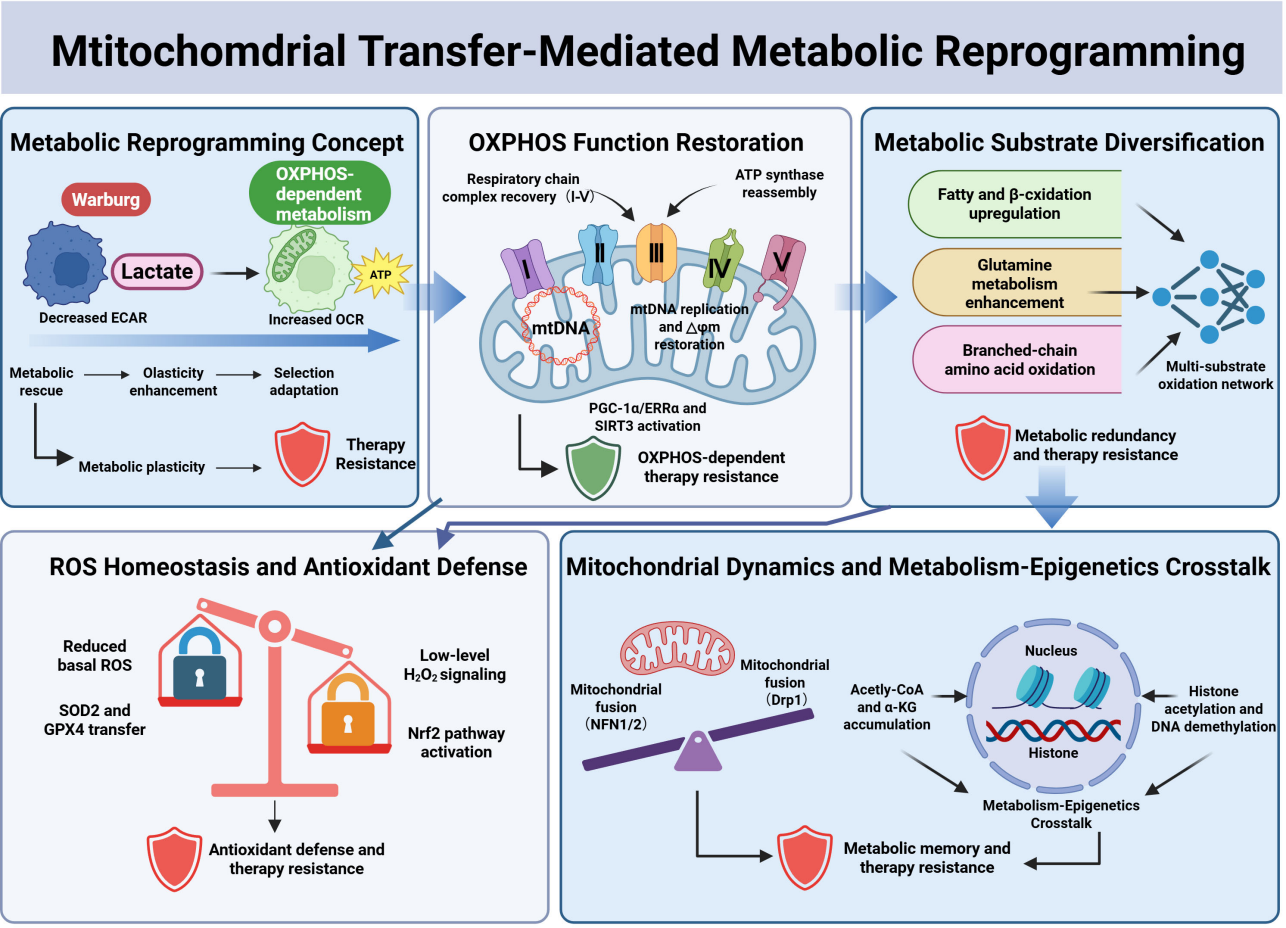
Overview of mitochondrial transfer-mediated metabolic reprogramming in bone metastatic tumor cells. This schematic summarizes the five key dimensions of metabolic reprogramming induced by intercellular mitochondrial transfer. Upper left panel (Metabolic Reprogramming Concept): Mitochondrial transfer drives a transition from the glycolysis-dominant Warburg phenotype to an OXPHOS-dependent metabolic state, characterized by decreased ECAR and increased OCR. This transition follows a three-stage progression—metabolic rescue, plasticity enhancement, and selective adaptation—ultimately contributing to enhanced metabolic plasticity and therapy resistance. Upper middle panel (OXPHOS Function Restoration): Transferred mitochondria restore respiratory chain complex (I–V) activity, initiate mtDNA replication and membrane potential (ΔΨm) recovery, and complete ATP synthase reassembly. Activation of the PGC-1α/ERRα transcriptional complex and SIRT3-mediated deacetylation further consolidate OXPHOS-dependent therapy resistance. Upper right panel (Metabolic Substrate Diversification): Following transfer, tumor cells establish a multi-substrate oxidation network through upregulation of fatty acid β-oxidation, enhancement of glutamine metabolism, and activation of branched-chain amino acid oxidation, collectively conferring metabolic redundancy and therapy resistance. Lower left panel (ROS Homeostasis and Antioxidant Defense): Co-transferred antioxidant enzymes (SOD2 and GPX4) reduce basal ROS levels while maintaining low-level H_2_O_2_ signaling, which activates the Nrf2 pathway and strengthens antioxidant defense, contributing to therapy resistance. Lower right panel (Mitochondrial Dynamics and Metabolism–Epigenetics Crosstalk): Transferred mitochondria promote MFN1/2-mediated mitochondrial fusion while suppressing Drp1-dependent fission. Accompanying accumulation of acetyl-CoA and α-KG drives histone acetylation and DNA demethylation through metabolism–epigenetics crosstalk, establishing a “metabolic memory” mechanism that sustains long-term therapy resistance.

Physicochemical features of the microenvironment also participate in this regulation: hypoxic conditions (1% O_2_) substantially increase transfer efficiency through the HIF-1α/Miro1 signaling axis (see Section 2.2) (113); calcium ion gradients enhance Miro1-mediated mitochondrial transport via the MCU (114); and an acidic microenvironment (pH ~6.5) upregulates the expression of TNT-associated markers through G protein-coupled receptor 65 (GPR65) (**Figure 2**) (115).

In addition, anti-tumor therapies can reshape transfer dynamics. ATP efflux induced by chemotherapeutic agents activates the purinergic receptor P2Y2 on the BMSC surface, promoting directional TNT growth (116). CDK4/6 inhibitors, by arresting the cell cycle at G1 phase, may extend the time window during which transfer can occur (117). Complex interactions likely exist among these multiple factors (118), and combined intervention strategies simultaneously targeting donor cells, recipient cells, and the microenvironment may produce synergistic effects, though direct experimental validation of such multi-target combinations in bone metastasis settings is still limited.

## 5 Mitochondrial transfer-mediated metabolic reprogramming

### 5.1 Conceptual framework of metabolic reprogramming

The mechanistic descriptions in this chapter integrate evidence from heterogeneous experimental systems, including breast cancer cell lines (e.g., MCF-7), hematologic malignancy models, and *in vitro* co-culture studies. Direct *in vivo* validation in solid-tumor bone metastatic specimens remains limited for most of the molecular events discussed below, and the reader should interpret individual mechanistic claims with the corresponding source system in mind.

Metabolic reprogramming refers to the adaptive alteration of metabolic pathways in response to changing physiological or pathological conditions. Metabolic reprogramming associated with mitochondrial transfer has been described as a transition of tumor cells from a glycolysis-dominant Warburg phenotype to an OXPHOS-dependent or mixed metabolic state. Seahorse metabolic analysis has reported that breast cancer cells receiving BMSC-derived mitochondria in co-culture systems show an increase in OCR accompanied by a decrease in extracellular acidification rate (ECAR), with the OCR/ECAR ratio recovering toward levels seen in non-malignant cells. This transition has been proposed to confer enhanced metabolic resilience on tumor cells under conditions of nutrient restriction and therapeutic pressure (19), although *in vivo* validation in human bone metastatic specimens remains limited.

At the molecular level, transferred mitochondria restore respiratory chain function in recipient cells and promote mitochondrial biogenesis(see Section 5.2) (119). The pattern of metabolic substrate utilization also shifts accordingly, with fatty acids and glutamine replacing glucose as the primary oxidative substrates (see Section 5.3) (120). This process has been conceptualized as a three-stage progression of “metabolic rescue — plasticity enhancement — selective adaptation”: the early stage involves restoration of ATP homeostasis, the intermediate stage may support biosynthetic demands, and the late stage may result in the selection of resistant clones (121). This staged model is a useful conceptual framework but is largely inferential, drawn from cross-sectional observations in different experimental systems rather than from direct longitudinal tracking of the same tumor population over time. Single-cell metabolomics analysis has reported that tumor cells may differentiate into OXPHOS-highly dependent subpopulations following transfer, with such subpopulations showing association with post-chemotherapy survival in selected studies (122). The dual-track metabolic configuration in which glycolysis and OXPHOS operate in parallel, proposed to be established through mitochondrial transfer, may partially account for the therapeutic resistance observed in bone metastatic tumors. These observations suggest that precisely targeting the initial window of OXPHOS restoration may represent a potential intervention strategy, although such timing-based interventions have not yet been clinically tested.

### 5.2 Molecular mechanisms of OXPHOS functional restoration

The primary effect of mitochondrial transfer has been proposed to lie in the restoration of respiratory chain function. Blue native polyacrylamide gel electrophoresis (BN-PAGE) analyses have reported that transferred mitochondria carry intact respiratory chain supercomplexes. Complex I–IV activities have been reported to be rapidly restored, with upregulation of the 13 respiratory chain proteins encoded by mtDNA and improvement of electron transfer efficiency (123). The kinetics and completeness of this restoration vary across studies and depend strongly on the experimental system used.

**Figure 3:**
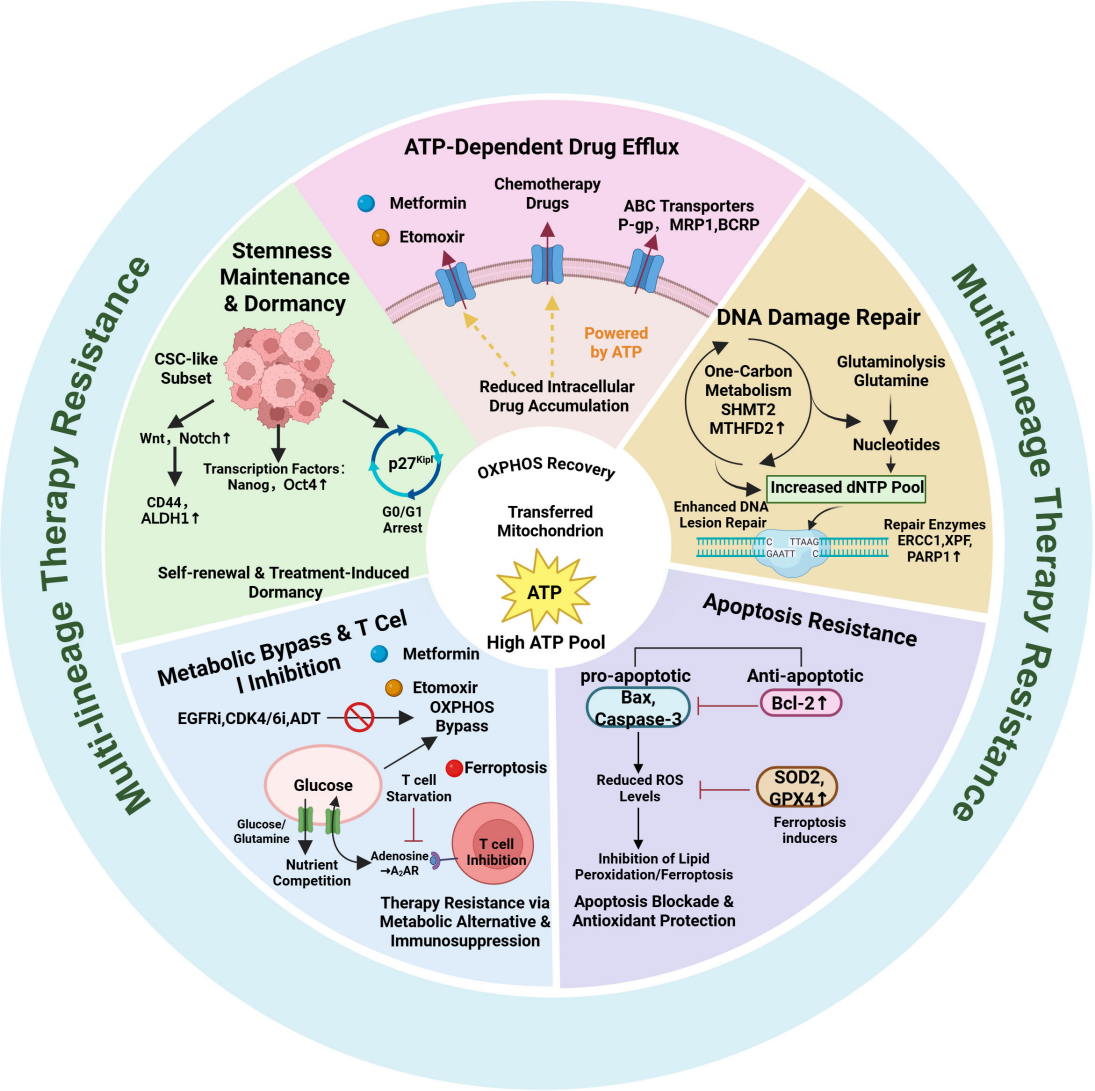
Molecular mechanisms of metabolic reprogramming-driven multi-lineage therapy resistance in bone metastatic tumor cells. This schematic illustrates how OXPHOS recovery following mitochondrial transfer generates a high ATP pool that drives five interconnected resistance mechanisms, collectively conferring multi-lineage therapy resistance. Central hub: Transferred mitochondria restore OXPHOS function and establish a high intracellular ATP pool, which serves as the energetic foundation for downstream resistance pathways. ATP-Dependent Drug Efflux (top): Elevated ATP powers ABC transporters (P-gp, MRP1, BCRP), reducing intracellular chemotherapy drug accumulation. Metformin is shown as a potential OXPHOS inhibitor to counteract this mechanism. DNA Damage Repair (upper right): One-carbon metabolism (SHMT2, MTHFD2) and glutaminolysis expand the dNTP pool, while upregulated repair enzymes (ERCC1, XPF, PARP1) enhance DNA lesion repair capacity. Apoptosis Resistance (lower right): Bcl-2 upregulation suppresses pro-apoptotic Bax/caspase-3 signaling, while co-transferred SOD2 and GPX4 reduce ROS levels and inhibit lipid peroxidation/ferroptosis, establishing antioxidant protection. Ferroptosis inducers are indicated as a potential countermeasure. Metabolic Bypass and T Cell Inhibition (lower left): OXPHOS bypass sustains energy supply during EGFRi, CDK4/6i, and ADT treatment. Tumor cells competitively deplete glucose and glutamine, causing T cell starvation, while adenosine signaling through A2AR further suppresses T cell function. Etomoxir is shown as a FAO inhibitor targeting the metabolic bypass. Stemness Maintenance and Dormancy (upper left): Activated Wnt and Notch pathways upregulate stemness transcription factors (Nanog, Oct4) and surface markers (CD44, ALDH1), maintaining a CSC-like subset. p27^Kip1-mediated G0/G1 arrest supports treatment-induced dormancy through OXPHOS-dependent basal metabolism. The outer ring emphasizes that these five mechanisms converge to generate multi-lineage therapy resistance encompassing chemotherapy, targeted therapy, endocrine therapy, and immunotherapy.

This restoration process has been proposed to involve several key molecular events. First, transferred mitochondria introduce functional mtDNA, which has been reported to activate DNA polymerase γ (POLG)-mediated replication fork formation and initiate mtDNA replication (124). Second, the high membrane potential (ΔΨm) of transferred mitochondria has been reported to diffuse through the voltage-dependent anion channel (VDAC), with the proposal that this may drive reversal of the depolarized state of endogenous mitochondria (125). Third, ATP synthase assembly factor 1/2 (ATPAF1/2) chaperones have been reported to facilitate F_0_F_1_ complex assembly, completing ATP synthase reassembly (126) (**Figure 3**).

Concurrently, the transcriptional regulatory network undergoes reprogramming. PGC-1α forms a transcriptional complex with estrogen-related receptor α (ERRα), upregulating nuclear respiratory factor 1/2 (NRF1/2) target genes to drive nuclear-encoded respiratory chain gene expression (106). Sirtuin 3 (SIRT3) promotes electron transfer by deacetylating and activating the NADH:ubiquinone oxidoreductase subunit A9 (NDUFA9) subunit of complex I (127), and SIRT3 protein carried by transferred mitochondria can directly act on the mitochondrial proteome of recipient cells (128). Functional validation has reported that post-transfer cells show partially restored sensitivity to oligomycin and rotenone, which has been interpreted as evidence for re-established OXPHOS dependence.

Respiratory chain supercomplex restoration, mtDNA replication initiation, and membrane potential re-establishment have been proposed to constitute the core mechanisms of OXPHOS functional reconstruction. Complex I, as the entry point of the electron transport chain, has been suggested as a candidate target for early intervention. However, much of this molecular detail derives from individual mechanistic studies; whether the entire chain of events occurs coherently in human bone metastatic lesions remains to be confirmed.

### 5.3 Diversification of metabolic substrate utilization

Mitochondrial transfer has been reported to broaden the metabolic substrate spectrum of tumor cells. Multiple isotope tracing studies have confirmed that, following transfer, glucose is primarily directed into the tricarboxylic acid (TCA) cycle rather than the lactate fermentation pathway, lactate output is reduced, and FAO flux is substantially enhanced, becoming the primary source of ATP production (129).

At the level of fatty acid metabolism, CPT1A and acyl-CoA dehydrogenase very long chain (ACADVL) expression is upregulated. Transferred mitochondria preferentially oxidize exogenous fatty acids, and fatty acid binding proteins co-transferred from BMSCs can sustain continuous FAO (130). Glutamine metabolism is also substantially enhanced: restored glutaminase 1 (GLS1) activity allows α-ketoglutarate (α-KG) to enter the TCA cycle directly, bypassing the pyruvate dehydrogenase (PDH) bottleneck (131). GLS1 protein carried by transferred mitochondria can directly activate glutaminolysis in recipient cells (132), though this observation derives from a small number of mechanistic reports and awaits independent validation.

Regarding amino acid metabolism, branched-chain amino acids (BCAAs) are oxidized through the branched-chain α-keto acid dehydrogenase (BCKDH) complex, a pathway that shows selective amplification in bone metastatic clones (133). Serine hydroxymethyltransferase 2 (SHMT2) expression is upregulated, activating one-carbon metabolism to support nucleotide synthesis (134).Pyruvate is redirected from the lactate pathway to OXPHOS following its repositioning to the inner mitochondrial membrane via mitochondrial pyruvate carrier 1 (MPC1) (135).

These changes have been proposed to form a “substrate stratification” pattern, in which fatty acids, glutamine, BCAAs, and glucose may collectively constitute a hierarchical energy supply system. The relative quantitative contribution of each substrate to *in vivo* tumor metabolism in bone metastatic lesions remains to be systematically quantified, and most current evidence derives from *in vitro* flux analyses that do not fully recapitulate the bone marrow microenvironment. Nevertheless, this multi-substrate oxidation network has been proposed to confer metabolic redundancy on bone metastatic tumors, which may partially account for the limited efficacy of single metabolic inhibitors observed in preclinical studies.

The current evidence supports the concept that mitochondrial transfer broadens substrate utilization in recipient tumor cells, but the strength of evidence varies considerably across substrates: FAO restoration has the strongest experimental support (multiple independent studies, isotope tracing evidence), glutamine pathway reprogramming has moderate support (primarily mechanistic studies in selected cell lines), and BCAA pathway involvement remains based on a limited number of reports. Combined FAO and glutaminolysis inhibition has been proposed as a more rational therapeutic strategy than single-pathway targeting; this hypothesis warrants direct testing in bone metastasis–specific preclinical models before clinical translation can be reasonably considered.

In bone metastasis, mitochondrial metabolic reprogramming predominantly involves enhanced TCA cycle flux, restored ETC/OXPHOS activity, and upregulated fatty acid β-oxidation, together with amino acid metabolism—particularly glutamine-fueled anaplerosis and branched-chain amino acid oxidation—supporting biosynthetic and bioenergetic demands (136). Following mitochondrial transfer, this rewiring is most consistently reflected in the recovery of OXPHOS capacity and TCA cycle activity in recipient tumor cells (137). By contrast, evidence for transfer-specific remodeling of the urea cycle, heme biosynthesis, the malate–aspartate shuttle, gluconeogenesis, and mitochondrial calcium homeostasis in bone metastasis remains limited and largely inferential, representing an important direction for future investigation (138, 139).

### 5.4 Effects of mitochondrial transfer on ROS homeostasis and antioxidant defense

Mitochondrial transfer has been proposed to reshape the redox homeostasis of tumor cells. Several studies have reported that healthy transferred mitochondria may reduce baseline ROS levels in recipient cells while preserving physiological ROS concentrations required for signaling functions. Antioxidant enzymes including SOD2 and glutathione peroxidase 4 (GPX4) are co-transferred with mitochondria, establishing a relatively complete defense system (27).

At the level of ROS regulation, transferred mitochondria reduce superoxide anion leakage through efficient electron transfer at complex II/III. SOD2 converts residual superoxide anions to hydrogen peroxide (H_2_O_2_) (140). H_2_O_2_ at concentrations of 50–100 nM can activate phosphoinositide 3-kinase/protein kinase B (PI3K/Akt) survival signaling, whereas high concentrations induce apoptosis (141, 142), the precise concentration thresholds, however, are likely context-dependent and may not be directly extrapolable across tumor types or microenvironments.

This low-level H_2_O_2_ simultaneously promotes Kelch-like ECH-associated protein 1–nuclear factor erythroid 2-related factor 2 (Keap1–Nrf2) dissociation, driving the transcriptional upregulation of phase II detoxification enzymes such as heme oxygenase-1 (HO-1) and NAD(P)H quinone dehydrogenase 1 (NQO1) (115). Nrf2 knockout has been reported to reduce transfer efficiency in selected studies (143), raising the possibility of a positive feedback loop, though this relationship requires further mechanistic dissection and has not yet been demonstrated in bone metastasis–specific *in vivo* models.

Optimization of ROS homeostasis is directly linked to chemotherapy tolerance: tumor cells exposed to ROS-inducing agents (such as anthracyclines) after transfer exhibit substantially reduced intracellular peak ROS concentrations, accompanied by decreased caspase-3 cleavage (144). This proposed functional shift of ROS — from a cytotoxic factor to a signaling molecule — may partially account for the antioxidant advantage observed in cells receiving mitochondrial transfer in preclinical systems.

Evidence for ROS modulation following mitochondrial transfer is mechanistically plausible but largely based on bulk-cell assays in co-culture systems; high-resolution measurements of ROS dynamics in individual recipient cells *in vivo* are technically challenging and remain limited. The hypothesis of a positive Nrf2–transfer feedback loop is intriguing but currently supported by a small number of studies. The proposed combination of Nrf2 inhibitors with OXPHOS inhibitors as a dual intervention strategy is conceptually attractive but has not been validated in bone metastasis preclinical models; potential off-target effects on normal Nrf2-dependent tissues (e.g., hematopoietic stem cells in the same bone marrow microenvironment) warrant particular caution.

### 5.5 Mitochondrial dynamics and metabolic–epigenetic crosstalk

Mitochondrial transfer can reshape the mitochondrial dynamics balance in recipient cells. Studies have shown that transferred mitochondria activate MFN1/2-mediated fusion, promoting a transition of the mitochondrial network from a fragmented to a tubular morphology to support efficient OXPHOS. Concurrently, decreased dynamin-related protein 1 (Drp1) phosphorylation levels suppress mitochondrial fission (145, 146).

Accompanying the remodeling of mitochondrial dynamics, metabolic–epigenetic crosstalk is also activated. Expansion of the acetyl-coenzyme A (acetyl-CoA) pool promotes increased histone H3 lysine 27 acetylation (H3K27ac) marks, activating metabolic gene cluster expression (147). α-KG mediates DNA demethylation through ten-eleven translocation (TET) enzymes, relieving the silencing of metabolism-related genes (148). Restoration of nicotinamide adenine dinucleotide (NAD^+^) levels activates SIRT1/3-mediated deacetylation, regulating the transcriptional activity of PGC-1α and forkhead box O3 (FOXO3) (149).

Lactate has also been proposed to function as an epigenetic signaling molecule, promoting H3K27ac deposition through inhibition of histone deacetylase (HDAC) enzymatic activity (150). Recent studies have reported that mtDNA heteroplasmy introduced through transfer may maintain epigenetic memory via metabolite signaling, with cells carrying mixed mtDNA retaining the OXPHOS phenotype across multiple generations in some experimental systems (151). These findings come from a limited number of studies and the concept of stable “metabolic memory” mediated by mtDNA heteroplasmy remains exploratory; direct evidence in bone metastasis settings has not been reported.

These observations have been proposed to constitute a “metabolic memory” mechanism that may contribute to longer-term maintenance of the metabolic advantage following mitochondrial transfer. It should be emphasized, however, that the dual remodeling of mitochondrial dynamics and epigenetic reprogramming is described primarily as a conceptual framework integrating findings from disparate experimental systems, rather than as a directly demonstrated coherent mechanism in any single *in vivo* model.

This section represents one of the more speculative areas within the mitochondrial transfer literature. The individual molecular events (MFN1/2-mediated fusion, α-KG–TET–DNA demethylation, NAD^+^–SIRT signaling) are well-established in general cell biology, but their specific operation downstream of mitochondrial transfer in bone metastatic tumor cells is supported predominantly by inference and a limited number of mechanistic studies. The proposed therapeutic strategy of HDAC inhibitors combined with mitochondrial dynamics interventions is conceptually plausible but currently lacks direct experimental evidence in bone metastasis models. Future studies will need to distinguish whether epigenetic changes observed after mitochondrial transfer represent a causal mechanism of sustained metabolic adaptation or a secondary consequence of altered metabolite pools.

## 6 Molecular mechanisms of metabolic reprogramming-driven drug resistance

The mechanisms discussed in this chapter should not be regarded as parallel, independent resistance pathways. Rather, they appear to operate at different hierarchical levels: direct biochemical mechanisms (ATP-dependent drug efflux, DNA damage repair) at the molecular level; adaptive mechanisms (apoptosis resistance, redox protection) at the cellular level; and integrated network mechanisms (targeted therapy and immunotherapy resistance, stemness and dormancy) at the tumor-microenvironment level. The strength of evidence varies considerably across these mechanisms — strongest for ATP-dependent drug efflux in hematologic malignancy models, weakest for the proposed link between mitochondrial transfer and immunotherapy resistance in solid-tumor bone metastasis. The discussion below should be read with this evidence hierarchy in mind.

### 6.1 Enhanced ATP-dependent drug efflux

The OXPHOS function restored through mitochondrial transfer (see Section 5.2) provides the energy foundation for ATP-dependent drug efflux pumps. In breast cancer cells that have received BMSC-derived mitochondria, expression of ATP-binding cassette (ABC) transporter family members, including P-glycoprotein (P-gp), multidrug resistance-associated protein 1 (MRP1), and breast cancer resistance protein (BCRP), is substantially upregulated. Elevated intracellular ATP concentrations have been associated with enhanced drug efflux flux in these experimental systems, although direct causal demonstration *in vivo* within bone metastatic lesions remains limited (152).

At the transcriptional level, HIF-1α activated by transferred mitochondria can directly bind to the P-gp gene promoter to enhance its transcription (153), while sterol regulatory element-binding protein 1 (SREBP1) regulates MRP1 expression through the metabolic intermediate citrate (154). *In vivo* imaging studies in mouse models have reported that intracellular accumulation of anthracycline drugs is reduced in tumor cells following transfer, accompanied by suppressed cytochrome c release (155).

Analysis of clinical specimens has further validated the relevance of this mechanism: P-gp expression levels in tumor tissues from patients with bone metastasis show a positive correlation with mtDNA heteroplasmy (156), a finding that is compatible with but does not directly prove a mechanistic link between mitochondrial transfer and drug efflux capacity in patients. Regarding intervention strategies, metformin, as an OXPHOS-targeting agent, in combination with venetoclax has been reported to reverse this resistance phenotype in preclinical models (157), though clinical translation specifically targeting bone metastatic disease has not been reported.

These observations support the hypothesis that active drug efflux driven by ATP supply may constitute one energy-dependent component of chemotherapy resistance in bone metastasis. Targeting OXPHOS to attenuate ATP supply has been proposed as a potential chemosensitization approach, but the bone marrow is itself an OXPHOS-dependent tissue (for both hematopoiesis and stromal function), and the therapeutic window for selective targeting requires careful evaluation.

### 6.2 Enhanced DNA damage repair capacity

Metabolic reprogramming associated with mitochondrial transfer has been reported to enhance damage repair capacity in tumor cells in preclinical models, through activation of nucleotide synthesis pathways and upregulation of DNA repair enzyme expression. At the level of nucleotide supply, transferred mitochondria have been reported to activate expression of SHMT2 and methylenetetrahydrofolate dehydrogenase 2 (MTHFD2) in the one-carbon metabolic cycle, expanding the deoxynucleoside triphosphate (dNTP) pool to support DNA polymerase activity (158). Glutamine catabolism has been reported to provide purine and pyrimidine precursors, which may bypass chemotherapy-induced nucleotide depletion (159).

At the level of repair pathways, activation of nucleotide excision repair (NER) constitutes the core mechanism. α-KG (whose epigenetic regulatory function was described in Section 5.5) mediates histone H3 lysine 9 (H3K9) demethylation through lysine demethylase 4B (KDM4B), relieving the transcriptional repression of excision repair cross-complementation group 1 (ERCC1) and xeroderma pigmentosum complementation group F (XPF) genes (148). Poly(ADP-ribose) polymerase 1 (PARP1) enhances single-strand break repair capacity in a manner dependent on nicotinamide phosphoribosyltransferase (NAMPT) upregulation (160). Comet assay validation has shown that the rate of DNA interstrand crosslink repair induced by platinum-based agents is substantially accelerated in tumor cells following transfer (161).

Enhancement of homologous recombination (HR) repair has also been linked to PARP inhibitor resistance. NAD^+^ levels restored by transferred mitochondria have been reported to activate SIRT1, which may promote breast cancer type 1 susceptibility protein (BRCA1) deacetylation and assembly of the HR complex (162). The mechanistic chain — mitochondrial transfer → NAD^+^ → SIRT1 → BRCA1 deacetylation → HRR repair — combines several individually plausible elements, but coherent demonstration of this entire chain in bone metastatic tumors has not yet been reported.

Evidence in this section is stronger for the upstream metabolic events (nucleotide pool expansion, glutamine-derived precursor supply) than for the downstream repair-pathway activation, which is supported predominantly by mechanistic plausibility and selected studies. The proposed combination of glutaminase inhibitors with PARP inhibitors as a rational strategy for overcoming this type of resistance is biologically reasonable but has not been tested in bone metastasis–specific clinical settings, and the systemic effects of glutaminase inhibition on normal tissue function (particularly in immune cells that also depend on glutamine) require further evaluation.

### 6.3 Apoptosis pathway resistance and antioxidant protection

Mitochondrial transfer blocks the mitochondrial apoptosis pathway by reshaping the balance of B-cell lymphoma 2 (Bcl-2) family proteins. Studies have shown that transferred mitochondria restore intracellular glutathione (GSH) levels and upregulate Bcl-2 expression, suppressing the oligomerization of the pro-apoptotic protein Bcl-2-associated X protein (Bax) (163). SOD2 co-transferred with mitochondria substantially reduces cytochrome c release, blocking apoptotic protease-activating factor 1 (Apaf-1) apoptosome formation (164).

The establishment of apoptosis resistance depends on the activation of the antioxidant system. As described in Section 5.4, mitochondrial transfer reshapes ROS homeostasis and activates the Nrf2 pathway. In terms of apoptosis resistance, Nrf2 nuclear translocation upregulates GPX4 and the cystine/glutamate antiporter (xCT) expression to suppress the ferroptosis pathway (165). GPX4 protein carried by transferred mitochondria can directly inhibit phospholipid peroxidation reactions (166). The catalytic subunit of glutamate-cysteine ligase (GCL), activated by Nrf2, maintains reduced GSH levels (167).

The extrinsic apoptosis pathway is also suppressed. Following transfer, the expression of the death receptor Fas (also known as CD95) is downregulated in tumor cells, and death-inducing signaling complex (DISC) formation is impaired (168). Chemotherapy-induced TNF-related apoptosis-inducing ligand (TRAIL) signaling is blocked through upregulation of cellular FLICE-inhibitory protein (c-FLIP) (169).

The proposed dual suppression of intrinsic and extrinsic apoptosis pathways, combined with activation of antioxidant defense, has been suggested to collectively contribute to apoptosis resistance. It should be noted that most of these individual mechanisms have been demonstrated separately in different experimental systems; whether they operate coherently and simultaneously within the same recipient tumor cells *in vivo* remains to be established.

Based on these mechanistic features, Bcl-2 homology domain 3 (BH3) mimetics combined with ferroptosis inducers have been proposed as a potential strategy for overcoming mitochondrial transfer-mediated apoptosis resistance. However, this combination strategy has not yet been tested in bone metastasis–specific clinical trials, and the safety profile of dual apoptosis pathway activation in patients with bone marrow involvement (including effects on normal hematopoiesis) requires careful preclinical evaluation.

The evidence base for apoptosis resistance following mitochondrial transfer is mechanistically diverse but methodologically fragmented. Most studies focus on individual apoptosis regulators rather than the integrated apoptosis decision-making machinery. The therapeutic implication — combining BH3 mimetics with ferroptosis inducers — is theoretically appealing but represents an early-stage hypothesis that requires substantial preclinical development before translational consideration, particularly given the bone marrow context in which both apoptosis activation and ferroptosis induction may affect normal hematopoietic populations.

### 6.4 Metabolic basis of targeted therapy and immunotherapy resistance

Metabolic reprogramming induced by mitochondrial transfer can mediate resistance to various targeted therapies through activation of bypass pathways. EGFR–tyrosine kinase inhibitor (EGFR-TKI)-resistant lung cancer bone metastatic tumors rely on OXPHOS bypass pathways to maintain energy supply, in which respiratory chain complex I activity restored through mitochondrial transfer plays a critical role (170). In CDK4/6 inhibitor resistance, FAO provides energy compensation (171).

At the signaling level, Akt phosphorylation activated by transferred mitochondria sustains PI3K/Akt/mechanistic target of rapamycin (mTOR) proliferative signaling (172). The AMP-activated protein kinase (AMPK) energy-sensing pathway is suppressed to prevent metabolic stress signal transduction (173), and mesenchymal-epithelial transition factor (MET)-amplified resistant clones preferentially acquire mitochondria from BMSCs (174).

Endocrine therapy resistance likewise depends on metabolic bypass pathways. Following ADT, prostate cancer bone metastases maintain proliferation through FAO (175). Estrogen receptor (ER)-positive breast cancer endocrine resistance is accompanied by estrogen receptor 1 (ESR1) mutation and OXPHOS upregulation (176).

In the context of immunotherapy, high OXPHOS activity in bone metastatic tumors competitively depletes glucose and glutamine required by T cells, suppressing effector T cell function (170). Adenosine further inhibits T cell proliferation through the adenosine A2A receptor (A2AR) (55). It should be noted that the proposed mechanistic link between mitochondrial transfer and immunotherapy resistance is currently among the least developed in this review: most evidence is derived from general tumor immunometabolism studies rather than from direct investigation in bone metastatic disease, and direct experimental demonstration that mitochondrial transfer events reshape the immune response specifically in bone metastatic lesions is limited.

Metabolic reprogramming has also been associated with the maintenance of tumor stemness (see Section 6.5). These observations support the hypothesis that metabolic bypass activation and immune microenvironment remodeling may together contribute to the metabolic basis of targeted therapy and immunotherapy resistance. Combined OXPHOS inhibitors with immunometabolic modulators have been proposed as a potential strategy, though such combinations remain at an early experimental stage and have not been tested in randomized clinical trials specifically powered for bone metastasis outcomes.

This section integrates evidence from heterogeneous sources: targeted therapy resistance (relatively well-supported by preclinical data), endocrine resistance (supported by both preclinical and limited clinical observations), and immunotherapy resistance (largely inferential, based on extrapolation from immunometabolism principles to bone metastatic disease). The evidence hierarchy should be explicit in any translational discussion. The therapeutic concept of combining OXPHOS inhibitors with immune checkpoint inhibitors is currently best characterized as a hypothesis-generating direction rather than a treatment-ready strategy for bone metastasis.

### 6.5 Tumor stemness maintenance and dormancy

Mitochondrial transfer maintains the cancer stem cell (CSC) phenotype and supports therapy-induced dormancy through OXPHOS reprogramming. Studies have shown that breast cancer cells receiving BMSC-derived mitochondria exhibit substantial upregulation of CD44 and aldehyde dehydrogenase 1 (ALDH1), accompanied by transcriptional activation of Nanog, octamer-binding transcription factor 4 (Oct4), and sex-determining region Y-box 2 (Sox2). OXPHOS metabolism supports the self-renewal and asymmetric division of CSCs (5, 177).

At the signaling pathway level, β-catenin restored by transferred mitochondria maintains the stemness phenotype through the Wnt pathway (178). Enhanced Notch receptor internalization leads to Notch intracellular domain (NICD) nuclear translocation, which activates hairy and enhancer of split 1 (Hes1) to suppress differentiation gene expression (115). Hedgehog signaling maintains the stem cell niche through glioma-associated oncogene homolog 1 (Gli1) transcriptional activity (122). Following mitochondrial transfer, the CSC subpopulation exhibits a substantial survival advantage against doxorubicin and paclitaxel (155).

Regarding dormancy mechanisms, dormant cells in bone metastases show upregulated expression of cyclin-dependent kinase inhibitor p27^Kip1, entering G0 arrest and relying on OXPHOS rather than glycolysis to maintain basal metabolism (110). Transferred mitochondria provide minimal energy requirements to support long-term survival (179). Nrf2-mediated low ROS homeostasis (see Section 5.4) helps avoid oxidative stress-induced dormancy awakening (27).

Single-cell analysis has revealed that OXPHOS-high/stemness-high double-positive subpopulations dominate within bone metastatic lesions, and dormant clones maintain metabolic homeostasis through mitochondrial transfer (180). However, direct longitudinal evidence demonstrating that mitochondrial transfer events are required for dormancy maintenance — as opposed to being correlated with it — has not yet been established in patient-derived models.

The proposed OXPHOS-supported CSC maintenance and dormancy mechanisms may partially account for the late relapse characteristics of bone metastasis. Wnt/Notch inhibitors combined with OXPHOS blockers have been proposed as a potential strategy for reducing relapse from this resistant reservoir, though this concept currently rests primarily on biological plausibility rather than direct experimental validation in bone metastasis dormancy models.

**Figure 4:**
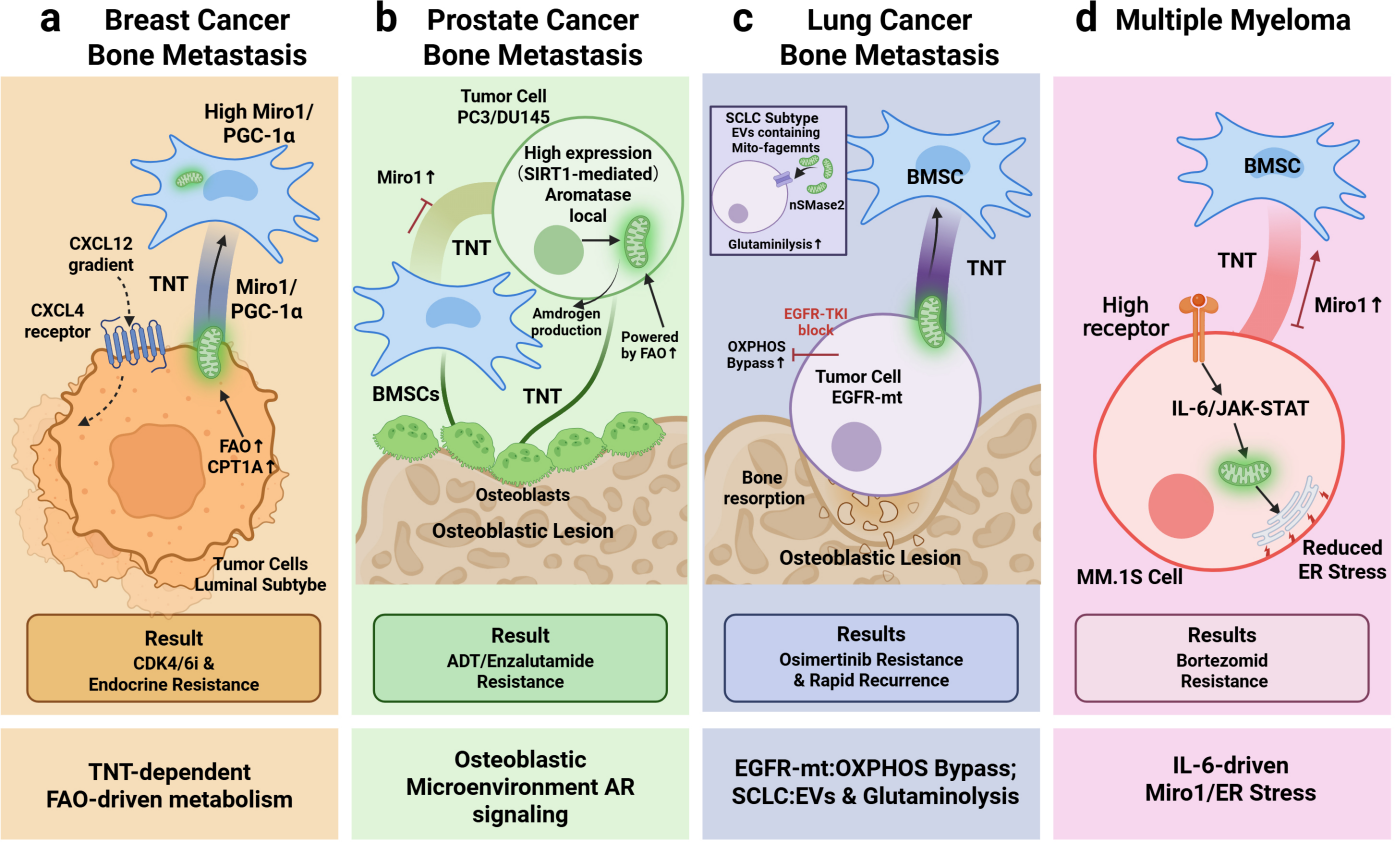
Tumor type-specific patterns of mitochondrial transfer and associated therapy resistance in bone metastasis. This schematic summarizes the distinct mitochondrial transfer mechanisms and resistance outcomes across four major tumor types with bone involvement. **(A)** Breast Cancer Bone Metastasis: In Luminal subtype tumor cells, BMSCs with high Miro1/PGC-1α expression transfer mitochondria via TNTs guided by the CXCL12/CXCR4 chemokine gradient. Transferred mitochondria enhance FAO flux through CPT1A upregulation, establishing a TNT-dependent, FAO-driven metabolic phenotype that contributes to CDK4/6 inhibitor and endocrine therapy resistance. **(B)** Prostate Cancer Bone Metastasis: Within the osteoblastic microenvironment, BMSCs and osteoblasts transfer mitochondria to PC3/DU145 tumor cells via high-density TNT networks. Miro1 upregulation facilitates transfer, while SIRT1-mediated AR deacetylation activates local aromatase expression and androgen production, powered by enhanced FAO. This osteoblastic microenvironment-AR signaling axis underlies ADT and enzalutamide resistance. **(C)** Lung Cancer Bone Metastasis: Two subtype-specific transfer patterns are depicted. In EGFR-mutant NSCLC, BMSCs transfer mitochondria via TNTs to tumor cells within osteoblastic lesions; restored OXPHOS function establishes a metabolic bypass that circumvents EGFR-TKI blockade, leading to osimertinib resistance. In the SCLC subtype (inset), tumor cells acquire mitochondrial fragments through nSMase2-mediated EVs, restoring glutaminolysis to support rapid recurrence. **(D)** Multiple Myeloma: BMSCs transfer mitochondria to MM.1S cells via TNTs with high Miro1 expression, driven by the IL-6/Janus kinase–signal transducer and activator of transcription (JAK-STAT) signaling axis. Transfer reduces endoplasmic reticulum stress in recipient myeloma cells, contributing to bortezomib resistance. Bottom panels summarize the defining mechanistic feature of each tumor type.

The intersection of mitochondrial transfer with CSC biology and dormancy represents one of the most clinically important — and most evidentially limited — areas of this field. The conceptual appeal is high: if mitochondrial transfer indeed sustains the dormant CSC reservoir responsible for late relapse in bone metastasis, then targeting this transfer could in principle reduce relapse risk. However, the experimental evidence directly linking transfer events to dormant cell survival *in vivo* is currently sparse, and most of the cited mechanistic pathways (Wnt, Notch, Hedgehog, p27, Nrf2) are general CSC/dormancy regulators rather than transfer-specific effectors. Distinguishing transfer-specific from transfer-independent components of CSC and dormancy biology will be essential before therapeutic strategies targeting this axis can be rationally developed (**Figure 4**).

## 7 Tumor type-specific analysis

The discussion in this chapter integrates evidence across four distinct biological settings: HR-positive and triple-negative breast cancer bone metastasis (osteolytic, with mixed evidence base), prostate cancer bone metastasis (predominantly osteoblastic, with selected preclinical and clinical evidence), lung cancer bone metastasis (least developed evidence base, primarily inferential), and multiple myeloma (marrow-resident primary malignancy, included as the most direct experimental reference rather than a true bone metastatic disease). The reader should bear in mind that direct *in vivo* evidence in solid-tumor bone metastasis is substantially less developed than evidence in MM or AML, and that conclusions drawn for solid tumors are frequently derived by extrapolation from hematologic malignancy findings or from co-culture systems.

### 7.1 Breast cancer bone metastasis

Recent studies have further clarified the mitochondrial bioenergetic basis of breast cancer heterogeneity and its relevance to therapy response. Breast cancer cells display pronounced mitochondrial heterogeneity, with subpopulations differing in respiratory chain supercomplex assembly, mtDNA copy number, and OXPHOS dependence, which underlies their differential metabolic plasticity and drug tolerance (181, 182). Metabolic rewiring toward enhanced OXPHOS and FAO has been consistently associated with endocrine and CDK4/6 inhibitor resistance, whereas mitochondrial-targeted strategies—including complex I inhibitors, FAO inhibitors, and agents disrupting mitochondrial biogenesis via the PGC-1α/ERRα axis—have shown preclinical activity in reversing these resistant phenotypes (183, 184). Against this background, mitochondrial transfer may be viewed as one upstream event that shifts recipient tumor cells toward the OXPHOS-high, metabolically plastic state associated with therapy resistance in breast cancer bone metastasis (185). Most of these mitochondrial-targeted agents, however, have been evaluated in primary or visceral disease rather than specifically in bone metastatic lesions.

Reports comparing mitochondrial transfer characteristics across molecular subtypes are emerging but remain limited in number; the subtype-specific patterns summarized below should be interpreted as preliminary observations rather than established mechanisms. A limited number of comparative studies have reported a higher frequency of mitochondrial transfer from BMSCs to HR-positive than to triple-negative breast cancer (TNBC) cells in preclinical models (186, 187). TNT-mediated transfer has been reported to restore OXPHOS function (see Section 5.2 for mechanisms) and to be associated with CDK4/6 inhibitor resistance in preclinical models (188).

Regarding subtype-specific mechanisms, Luminal A breast cancer relies on the CXCL12/CXCR4 axis (see Section 2.1) to induce TNT formation, with enhanced FAO flux following transfer (189). The triple-negative subtype, in contrast, maintains the CSC phenotype through glutamine catabolism, with activation of GLS1 expression (22). Single-cell metabolomics analysis has further confirmed the selective expansion of OXPHOS-highly active clones in breast cancer bone metastases (122), though the directionality — whether mitochondrial transfer drives OXPHOS clonal expansion or whether OXPHOS-high clones are preferentially selected by an OXPHOS-supportive microenvironment — remains incompletely resolved.

At the level of endocrine therapy resistance, transferred mitochondria can support the activation of the androgen receptor (AR) signaling pathway to maintain tumor proliferation following aromatase inhibitor (AI) resistance (190). However, the mechanistic link between mitochondrial transfer and AI resistance in HR-positive breast cancer bone metastasis is supported by a limited number of studies and has not yet been validated in patient-derived xenograft or clinical cohort settings.

These inter-subtype differences suggest that intervention strategies for breast cancer bone metastasis may need to consider molecular subtyping: Luminal subtypes may potentially benefit from CXCR4 axis blockade to attenuate mitochondrial transfer, whereas triple-negative subtypes may be more amenable to targeting the glutamine metabolic pathway. The clinical value of such differentiated strategies remains hypothetical and requires direct validation in subtype-specific clinical trials, which have not yet been conducted.

Breast cancer bone metastasis is the solid-tumor setting with the most developed mitochondrial transfer evidence base, but “most developed” should not be confused with “well-established.” Evidence is mostly derived from co-culture and mouse models; direct clinical evidence linking transfer events to therapy resistance in breast cancer bone metastasis patients is currently absent. The proposed subtype-specific intervention strategies are biologically reasonable but should be regarded as hypothesis-generating until tested in appropriate translational platforms.

### 7.2 Prostate cancer bone metastasis

Prostate cancer bone metastasis is characterized by osteoblastic lesions, and mitochondrial transfer plays an important role in maintaining androgen-independent growth. Studies have shown that PC3 and DU145 cells acquire mitochondria from BMSCs, leading to activation of aromatase expression and local androgen production that supports tumor survival. Transfer efficiency is negatively correlated with time to disease progression following ADT (191, 192). Whether this mechanism operates at a clinically significant level in patients with castration-resistant prostate cancer remains to be directly demonstrated.

The osteoblastic microenvironment further enhances mitochondrial transfer. Osteoblast-derived TNT networks exhibit higher density than those in osteoclast-dominated osteolytic metastatic lesions. CXCL12 gradients guide directional transfer (193) (see Section 2.1), and FAO serves as the primary energy pathway (see Section 5.3), with CPT1A maintaining elevated expression levels (194).

At the level of resistance mechanisms, NAD^+^ levels reportedly restored by transferred mitochondria have been reported to activate SIRT1 (the role of the sirtuin family in OXPHOS restoration was described in Section 5.2), which has been proposed to promote AR deacetylation, nuclear translocation, and transcriptional activity (195). Resistant clones to the second-generation AR antagonist enzalutamide have also been reported to rely on OXPHOS bypass pathways to maintain proliferation in preclinical models (196). These findings indicate that the osteoblastic microenvironment of prostate cancer bone metastasis provides favorable conditions for mitochondrial transfer. Inhibition of AR deacetylation combined with OXPHOS blockade may represent a potential combination strategy for overcoming castration-resistant prostate cancer (CRPC) resistance.

### 7.3 Lung cancer bone metastasis

The patterns of mitochondrial transfer in lung cancer bone metastasis have been proposed to relate to molecular subtype, though the evidence base in this setting is the least developed among the tumor types discussed in this review. In non-small cell lung cancer (NSCLC), mitochondrial transfer-associated resistance is particularly prominent in EGFR mutation-positive cases. In osimertinib-resistant bone metastatic lesions, tumor cells acquire mitochondria from BMSCs, and OXPHOS activation constitutes a metabolic bypass (see Section 5.2 for mechanisms and Section 6.4 for bypass resistance) (197, 198). EGFR wild-type lung cancer bone metastases rely on an indirect transfer mode mediated by the immune microenvironment, in which TAMs serve as intermediaries to form a tertiary transfer chain (see Section 4.2) (199).

Small cell lung cancer (SCLC) bone metastasis exhibits distinct features: tumor cells primarily acquire mitochondrial fragments through EVs and restore glutamine catabolism to support rapid relapse (200). TNT density within the bone marrow niche also increases substantially following chemotherapy (201). However, whether the predominant transfer mode in SCLC is genuinely EV-based — as opposed to TNT-based with EVs serving only as secondary routes — has not been systematically established, and the distinction between mitochondrial fragments and intact mitochondria in EV cargo (see Section 2.5) is particularly relevant in this context.

Overall, evidence regarding mitochondrial transfer in lung cancer bone metastasis is derived predominantly from *in vitro* systems or small animal cohorts; conclusions drawn for NSCLC and SCLC bone metastasis should therefore be interpreted with particular caution. Subtype-stratified intervention strategies have been proposed in principle — preferentially targeting OXPHOS bypass in EGFR-mutant tumors and the glutamine metabolic pathway in SCLC — but the clinical applicability of such precision strategies remains hypothetical, and has not been tested in lung cancer bone metastasis–specific clinical cohorts (202, 203).

Lung cancer bone metastasis represents an important but underdeveloped area within the mitochondrial transfer field. The proposed subtype-specific patterns — EGFR-mutant direct BMSC-to-tumor transfer, EGFR wild-type TAM-mediated cascade, and SCLC EV-dominant transfer — are individually plausible but rely on a limited number of primary studies, several of which have not been independently replicated. Substantial preclinical work in patient-derived models, including patient-derived xenograft and intratibial injection systems using primary patient tissue, is required before subtype-specific interventional strategies can be rationally proposed for clinical evaluation.

### 7.4 Multiple myeloma

Multiple myeloma occupies a position in this review that warrants explicit clarification at the outset. As discussed in Section 3.4, MM offers the most direct experimental access to BMSC–tumor mitochondrial transfer in a bone marrow context, and the mechanistic insights derived from MM have shaped much of the conceptual framework discussed in earlier sections. However, MM is biologically distinct from solid-tumor bone metastasis in fundamental ways: MM cells inhabit the marrow as their primary niche from disease onset, whereas metastatic solid tumors must first disseminate from a distant primary organ and colonize the marrow secondarily, undergoing distinct adaptation processes. Mechanistic findings in MM should therefore be regarded as informative — but not directly transferable — to the solid-tumor bone metastasis setting that constitutes the main focus of this review.

With these considerations in mind, MM has provided some of the most direct mechanistic evidence for mitochondrial transfer in a bone marrow context. As described in Section 4.1, MM.1S cells that acquire mitochondria from BMSCs have been reported to show restored OXPHOS flux and attenuated bortezomib-induced endoplasmic reticulum (ER) stress in co-culture and preclinical models (204, 205). Transfer events are directly associated with disease relapse risk (200), though the strength of this association in patient cohorts and its independence from other established prognostic factors remain to be systematically established (72, 206, 207).

Specific features of the bone marrow niche further enhance transfer efficiency. *In vivo* imaging has shown that TNT networks within the MM niche form high-density connection channels. The IL-6 signaling axis upregulates Miro1 expression (see Section 2.1 for Miro1 function), promoting mitochondrial transport (96, 208). Single-cell analysis has revealed that plasma cells can differentiate into an OXPHOS-dependent subtype following transfer (209, 210). It should be noted that these mechanistic observations, while internally consistent, have been derived predominantly from a small number of laboratories using related model systems; broader independent replication and validation in primary patient samples would strengthen the current evidence base.

Due to its origin within the bone marrow, MM provides an ideal model for investigating the clinical significance of mitochondrial transfer. Preliminary validation of proteasome inhibitor (PI) combined with OXPHOS blockade has provided initial support for the feasibility of this strategy. However, translation of MM-derived insights to solid-tumor bone metastasis should proceed with explicit recognition that the biological context is fundamentally different: solid-tumor bone metastatic cells have undergone an extensive dissemination and colonization adaptation process not shared by MM cells, the dominant donor cells and transfer routes may differ between osteolytic, osteoblastic, and primary marrow contexts (Section 3.4), and the relevant therapeutic vulnerabilities are unlikely to overlap completely. MM should be regarded in this review as a mechanistically informative comparator rather than as a parallel disease setting.

## 8 Therapeutic strategies targeting the mitochondrial transfer–metabolism axis

Therapeutic strategies targeting the mitochondrial transfer–metabolism axis can be conceptually approached from two perspectives: blocking the transfer process itself and interfering with the downstream metabolic consequences of transfer. The strategies summarized in this section are derived predominantly from preclinical investigations, and the clinical translation pathway for most of these approaches remains at an early stage. The reader should bear in mind throughout this section that no intervention specifically targeting mitochondrial transfer in bone metastasis has yet completed phase II clinical evaluation, and that several of the proposed agents have been tested mainly in hematologic malignancy contexts (see Section 6.4) rather than in solid-tumor bone metastasis. The discussion below is therefore framed as a conceptual roadmap for future translational development rather than as a description of established treatment options.

Regarding transfer blockade, cytochalasin D inhibits TNT formation by suppressing F-actin polymerization (211). The Miro1 small-molecule inhibitor Miro1-IN-1 specifically binds to the calcium-binding domain of Miro1 to block mitochondrial transport (212). GW4869 blocks the EV pathway through inhibition of nSMase2 (213). Multi-pathway combined blockade may overcome the compensatory risk associated with targeting a single pathway (214), though such combination approaches have not yet been validated in bone metastasis–specific *in vivo* systems.

Regarding metabolic intervention, OXPHOS inhibitors such as metformin and IACS-010759 can directly reverse the metabolic benefits conferred by mitochondrial transfer (215). Combination with the FAO inhibitor etomoxir can achieve systemic metabolic blockade. To reduce systemic toxicity, bone-targeted delivery systems—including bisphosphonate-conjugated nanocarriers, hydroxyapatite (HAp)-binding peptide-modified nanoparticles, and engineered EVs delivering Miro1 small interfering RNA (Miro1-siRNA)—have achieved selective enrichment within the bone microenvironment in preclinical models (216, 217).

Combination therapy strategies have validated the feasibility of multilevel intervention. Chemotherapy combined with OXPHOS inhibitors has substantially prolonged PFS (218). Targeted therapy combined with Miro1 inhibitors has improved the disease control rate (DCR) (219). Bone-targeted agents combined with metabolic intervention have reduced the incidence of SREs. Together, these approaches form a comprehensive strategic framework of “upstream blockade–midstream metabolic intervention–downstream bone protection.”

At the level of precision medicine, the establishment of multimodal biomarker panels—including mitochondrial uptake capacity of circulating tumor cells (CTCs), serum mtDNA heteroplasmy, and a three-gene signature comprising Miro1/PGC-1α/CPT1A—provides a basis for patient stratification and treatment response monitoring.

## 9 Challenges, controversies, and future directions

Conventional 2D co-culture systems do not adequately recapitulate the three-dimensional architecture, mechanical environment, oxygen gradients, and dynamic cellular composition of the bone marrow, and their transfer frequencies may not reflect physiological rates *in vivo* (Section 2.5). In TNT detection, fluorescent dye leakage and debris exchange are recognized sources of false-positive signals; because much of the early literature relied on dye-based methods, cross-study comparison requires careful methodological appraisal, and current *in vivo* tracing lacks the sensitivity to reliably capture low-frequency events. The central controversies in Section 2.5 remain unresolved—whether transferred material represents intact functional mitochondria or fragments, the durability of exogenous mitochondria within recipient cells, and the directionality and selectivity of transfer. Some studies report that transferred mitochondria fuse with the endogenous network and sustain respiration long-term, whereas others suggest a mainly short-term metabolic buffering role. Moreover, many pathways described here (Miro1-mediated TNT transport, nSMase2-dependent EV biogenesis, MFN1/2 fusion, Nrf2 antioxidant response) have been characterized predominantly in culture, and their quantitative contribution *in vivo* remains incompletely defined.

These uncertainties propagate into clinical translation. Insufficient local concentrations of Miro1 inhibitors in bone, dose-limiting systemic toxicity of OXPHOS inhibitors (Section 8), and the absence of long-term safety validation for bone-targeted nanodelivery limit the current pharmacological toolkit, while the lack of validated stratification biomarkers (Section 8) restricts enrichment of likely responders. A deeper challenge is demonstrating clinical benefit specifically attributable to mitochondrial transfer inhibition, as distinct from broader metabolic modulation, which will require trial designs and endpoints not yet established. The absence of any completed phase II trial targeting mitochondrial transfer in bone metastasis remains the principal barrier to clinical advancement. Future efforts must also address bone marrow–specific safety concerns—effects on normal hematopoiesis, bone remodeling, and immune function—which have not been systematically examined.Progress requires coordinated technical, mechanistic, and translational advances. Single-mitochondrion barcode CRISPR editing, *in vivo* two-photon microscopy with adaptive optics, spatial multi-omics (transcriptomics, metabolomics, mtDNA analysis), and genome-wide CRISPR screens could improve detection precision, resolve microenvironmental heterogeneity, and identify networks governing TNT formation, EV biogenesis, and post-transfer mitochondrial fate. These should be paired with patient-derived platforms—organoid bone metastasis models, intratibial xenografts from primary patient tissue, and standardized biobanks—to bridge mechanism and clinical relevance. Clinical translation should be staged and biomarker-driven: biomarker discovery and validation in independent cohorts, phase I dose-escalation trials in molecularly defined patients, retrospective subgroup analyses of OXPHOS inhibitors in existing trials, and pharmacokinetic/safety studies of bone-targeted nanocarriers.

Taken together, current evidence suggests that mitochondrial transfer does not merely restore baseline mitochondrial function in metastatic tumor cells but may actively reprogram mitochondrial fitness and signaling, thereby altering multiple interconnected cellular programs—energy homeostasis, redox balance, biosynthesis, and epigenetic regulation—that collectively promote survival and therapeutic resistance. At the pathway level, the metabolic rewiring following transfer appears to converge on enhanced TCA cycle flux, restored electron transport chain and OXPHOS activity, and upregulated FAO, with additional contributions from glutamine-fueled anaplerosis, branched-chain amino acid oxidation, and one-carbon metabolism supporting nucleotide synthesis; by contrast, direct evidence for transfer-specific remodeling of the urea cycle, heme biosynthesis, the malate–aspartate shuttle, gluconeogenesis, and mitochondrial calcium homeostasis in bone metastasis remains sparse and largely inferential. These metabolic adaptations are closely coupled to the bone-metastatic signaling milieu: RANKL and osteoprotegerin, as central regulators of the osteoclast–osteoblast axis, drive bone remodeling that liberates growth factors and increases metabolic substrate availability within the niche, while TGF-β and IGF-1 released from the bone matrix promote OXPHOS-dependent bioenergetics and mitochondrial biogenesis in recipient tumor cells. In this way, mitochondrial transfer and the surrounding signaling network act in concert to reinforce the metabolic fitness that underlies tumor-cell survival and therapeutic resistance.

Importantly, mitochondrial functional parameters—OXPHOS capacity, electron transport chain integrity, the proton gradient, and mitochondrial membrane potential (ΔΨm)—are governed not only by transferred mtDNA-encoded subunits but also by nuclear DNA–encoded regulators such as PGC-1α, ERRα, NRF1/2, TFAM, and the SIRT3 deacetylation network, and the coordinated interplay between transferred organelles and this nuclear regulatory machinery likely determines the durability of the resistant phenotype.

Finally, several context-dependent features warrant emphasis. First, a potential negative feedback loop may exist in which mitochondrial transfer lowers recipient-cell ROS through co-transferred antioxidant enzymes, while residual ROS itself acts as an upstream trigger for further transfer, creating a self-limiting yet resistance-promoting circuit. Second, under the hypoxic conditions characteristic of the bone marrow niche, transferred mitochondria may persist through HIF-1α–supported metabolic adaptation, sustaining OXPHOS-dependent survival even in low-oxygen microdomains. Third, these conditions are accompanied by distinct metabolic alterations, whereby recipient cells balance transferred OXPHOS capacity against hypoxia-driven glycolytic reliance, dynamically shifting substrate utilization to preserve bioenergetic and redox homeostasis. Each of these concepts, however, rests on a limited number of studies and requires direct validation in bone metastasis–specific *in vivo* systems.

Taken together, the field of mitochondrial transfer in bone metastasis stands at an intermediate stage of maturity, in which the foundational mechanistic concepts are increasingly well-supported in preclinical systems, the clinical relevance is biologically plausible but not yet directly demonstrated, and the translational pathway remains to be defined; its strength lies in the convergence of mechanistic interest, clinical need, and technological feasibility, whereas its limitations lie in the gap between elegant preclinical observations and rigorously validated clinical impact. Closing this gap will require sustained collaboration between basic researchers, translational scientists, and clinicians, with explicit attention to the controversies and evidence limitations identified throughout this review.

## Glossary

SREs: skeletal-related events
RANKL: receptor activator of nuclear factor-κB ligand
TGF-β: transforming growth factor-β
OXPHOS: oxidative phosphorylation
HR: hormone receptor
PFS: progression-free survival
BMSCs: bone marrow mesenchymal stem cells
TNTs: tunneling nanotubes
EVs: extracellular vesicles
Miro1: mitochondrial Rho GTPase 1
OCR: oxygen consumption rate
ATP: adenosine triphosphate
TNF-α: tumor necrosis factor-α
SOD2: superoxide dismutase 2
TOM20: translocase of the outer membrane 20
MFN1/2: mitofusin 1 and 2
PINK1: PTEN-induced kinase 1
FAO: fatty acid β-oxidation
MCU: mitochondrial calcium uniporter
ADT: androgen deprivation therapy
MM: multiple myeloma
CD36: cluster of differentiation 36
Tregs: regulatory T cells
EGFR: epidermal growth factor receptor
ECAR: extracellular acidification rate
ΔΨm: mitochondrial membrane potential
VDAC: voltage-dependent anion channel
ATPAF1/2: ATP synthase assembly factor 1/2
ERRα: estrogen-related receptorα
GPX4: glutathione peroxidase 4
H3K27ac: histone H3 lysine 27 acetylation
TET: ten-eleven translocation
SREBP1: sterol regulatory element-binding protein 1
MTHFD2: methylenetetrahydrofolate dehydrogenase 2
ERCC1: excision repair cross-complementation group 1
XPF: xeroderma pigmentosum complementation group F
NAMPT: nicotinamide phosphoribosyltransferase
BRCA1: breast cancer type 1 susceptibility protein
xCT: cystine/glutamate antiporter
GCL: glutamate-cysteine ligase
TRAIL: TNF-related apoptosis-inducing ligand
ER: estrogen receptor
ESR1: estrogen receptor 1.HRR, homologous recombination repair
Drp1: dynamin-related protein 1
NRF1/2: nuclear respiratory factor 1/2
SIRT3: sirtuin 3
TFAM: mitochondrial transcription factor A
CPT1A: carnitine palmitoyltransferase 1A
GLS1: glutaminase 1
PGC-1α: peroxisome proliferator-activated receptor γ
